# Supplementary figures and images for: The F1148 hydrophobic lock: A critical determinant of SARS-CoV-2 spike protein-mediated membrane fusion via the 3H/CH cavity
Source: PLoS Pathog. 2025 Sep 19;21(9):e1013526. doi: 10.1371/journal.ppat.1013526 (PMC12459827; doi:10.1371/journal.ppat.1013526)

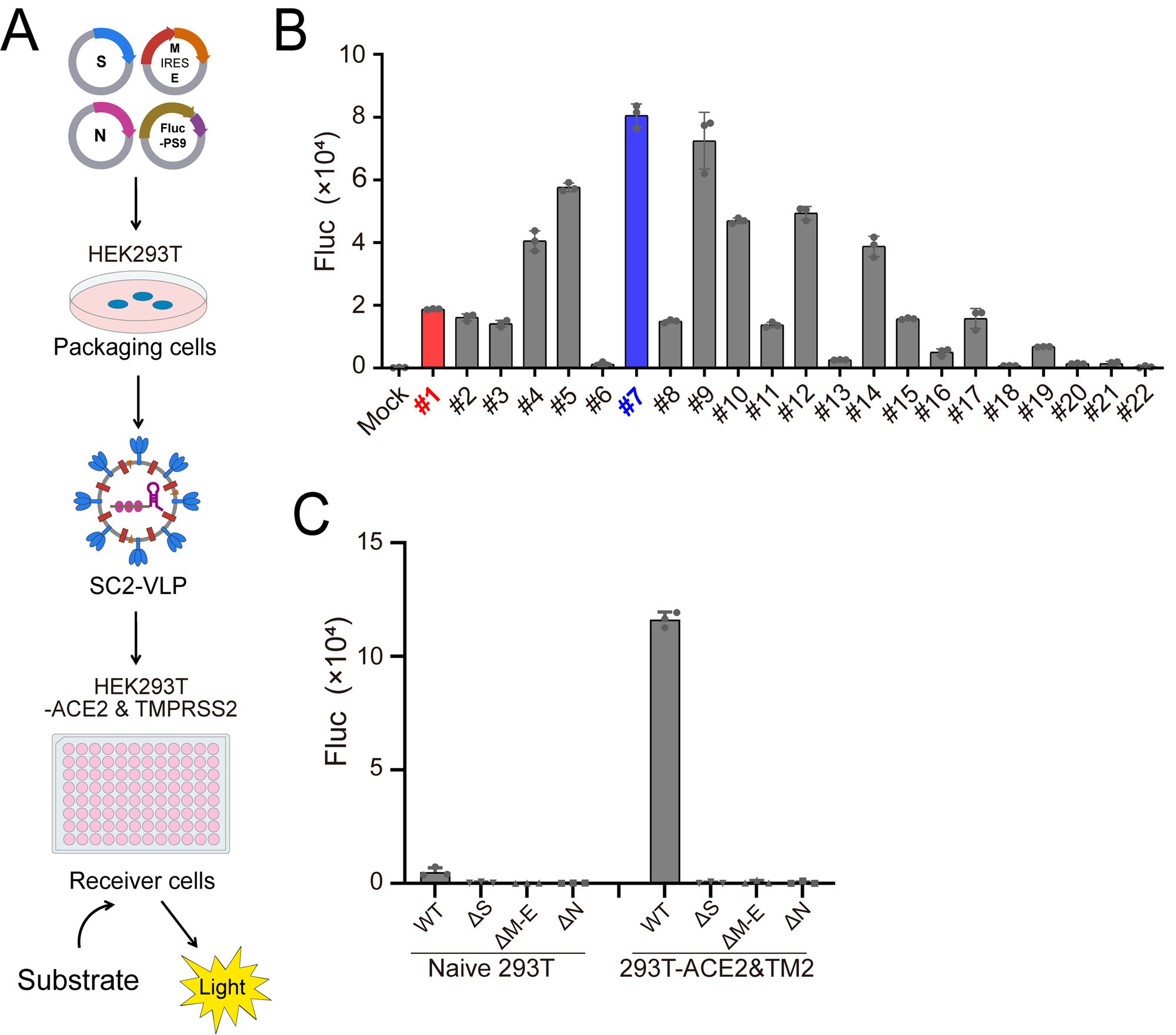

Supplement: S1 Fig — (A) Schematic of experiment design of the packaging and detection of SC2-VLP. SC2-VLP was generated by co-transfecting plasmids expressing Spike (S), membrane (M), envelope (E), nucleocapsid (N) and Fluc-PS9. The SC2-VLP were harvested and used to infect HEK293T cells co-expressing ACE2 and TMPRSS2. Infectivity was quantified by measuring Firefly luciferase activity 24 hours post-infection. (B) Uniform design-based optimization of plasmid transfection ratios. Following the principles of uniform design, different plasmid ratio groups were established for SC2-VLP packaging and HEK293T-ACE2&TMPRSS2 cell infection. Intracellular Firefly luciferase activities were determined at 24 hours post-infection (mean values ± SDs, n = 3). The initial condition [36] (#1, red) and optimized condition (#7, blue), are indicated. (C) Structural protein requirement for SC2-VLP infectivity. The packaged SC2-VLP was used to infect naive HEK293T and HEK293T-ACE2&TMPRSS2 cells, and intracellular Firefly luciferase activities were determined at 24 hours post-infection (mean values ± SDs, n = 3). (TIF) [file ppat.1013526.s001.tif]

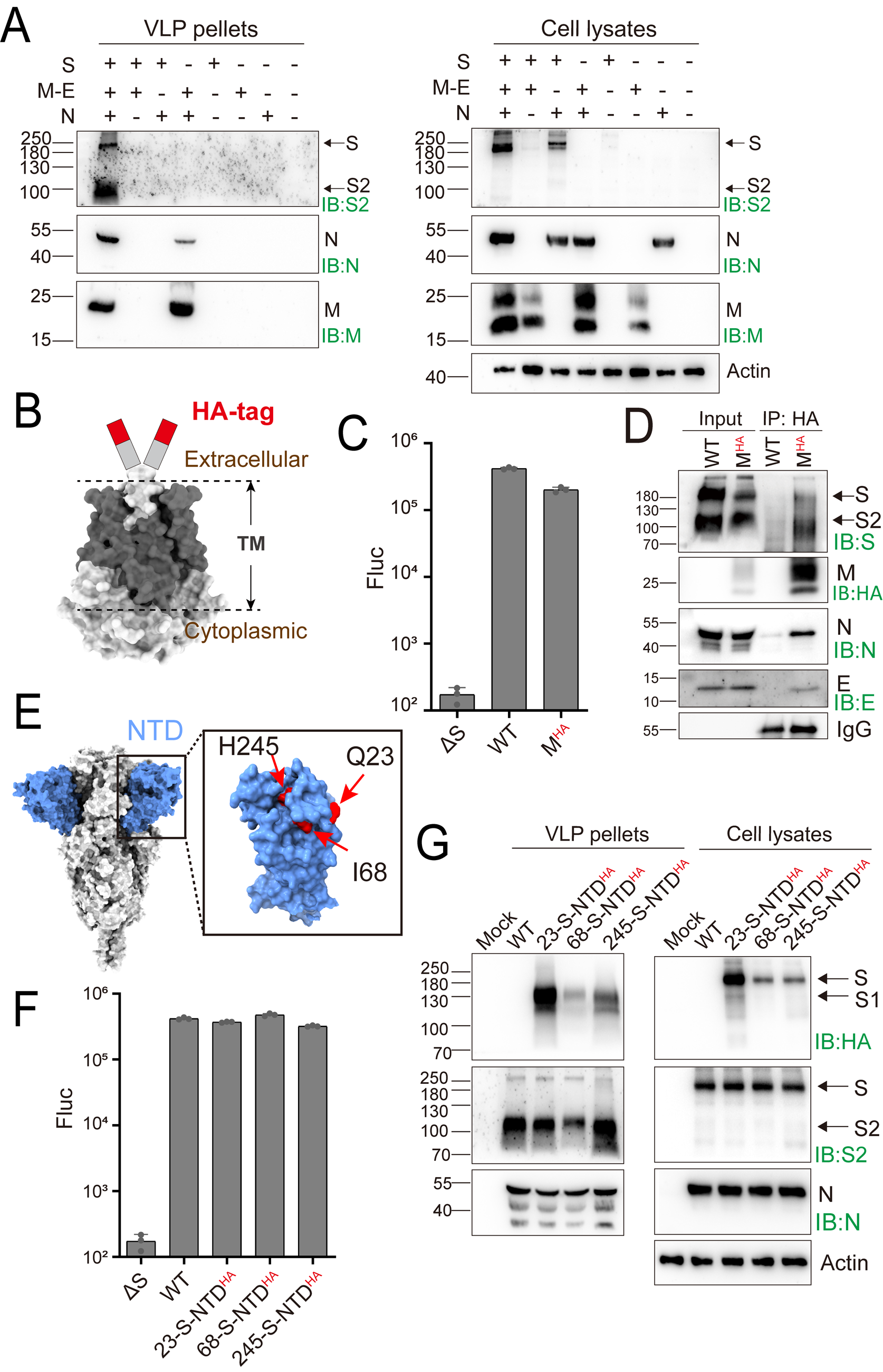

Supplement: S2 Fig — (A) Protein composition analysis of SC2-VLPs. Western blotting was performed using methanol-precipitated SC2-VLP supernatants and packaging cell lysates. Blots were probed with indicated antibodies (IB). The values to the left of the blots are molecular sizes in kilodaltons. The representative images shown for each group from multiple independent experiments.(B) Visualization of the HA-tag insertion site in the M structure. The HA-tag insertion site is located at the N-terminus of M, as depicted in the column cartoon representation of the unresolved structure (PDB ID: 7VGS). (C) Infectivity of SC2-VLP-MHA. HEK293T-ACE2/TMPRSS2 cells were infected with SC2-VLP-MHA, and intracellular Firefly luciferase activities were determined at 24 hours post-infection (mean values ± SDs, n = 3). (D) Immunoprecipitation of SC2-VLPs-MHA. HEK293T cells were transfected with plasmids to package SC2-VLPs-MHA, and the supernatant containing SC2-VLPs was collected 3 days post-transfection. Two aliquots were prepared: one was mixed with four volumes of methanol to precipitate the pelleted as input, while the other was incubated with anti-HA beads for protein capture (IP). The captured proteins were subjected to Western blotting assay with the indicated antibodies (IB), with representative images from three biological replicates are shown. The values to the left of the blots are molecular sizes in kilodaltons. (E) Structural visualization of the HA-tag insertion sites in the S NTD domain. The image was generated using PDB 6XR8. The enlarged region (boxed) indicated the residues of Q23, I68 and H245 (red) of the NTD domain (light blue). (F) The infectivity of HEK293T-ACE2&TMPRSS2 cells by SC2-VLPs-(S-NTDHA). The packaged SC2-VLPs-(S-NTDHA) was used to infect HEK293T-ACE2&TMPRSS2 cells, and intracellular Firefly luciferase activities were determined at 24 hours post-infection (mean values ± SDs, n = 3). (G) Western blotting of SC2-VLPs-(S-NTDHA). The SC2-VLP-containing supernatants precipita [file ppat.1013526.s002.tif]

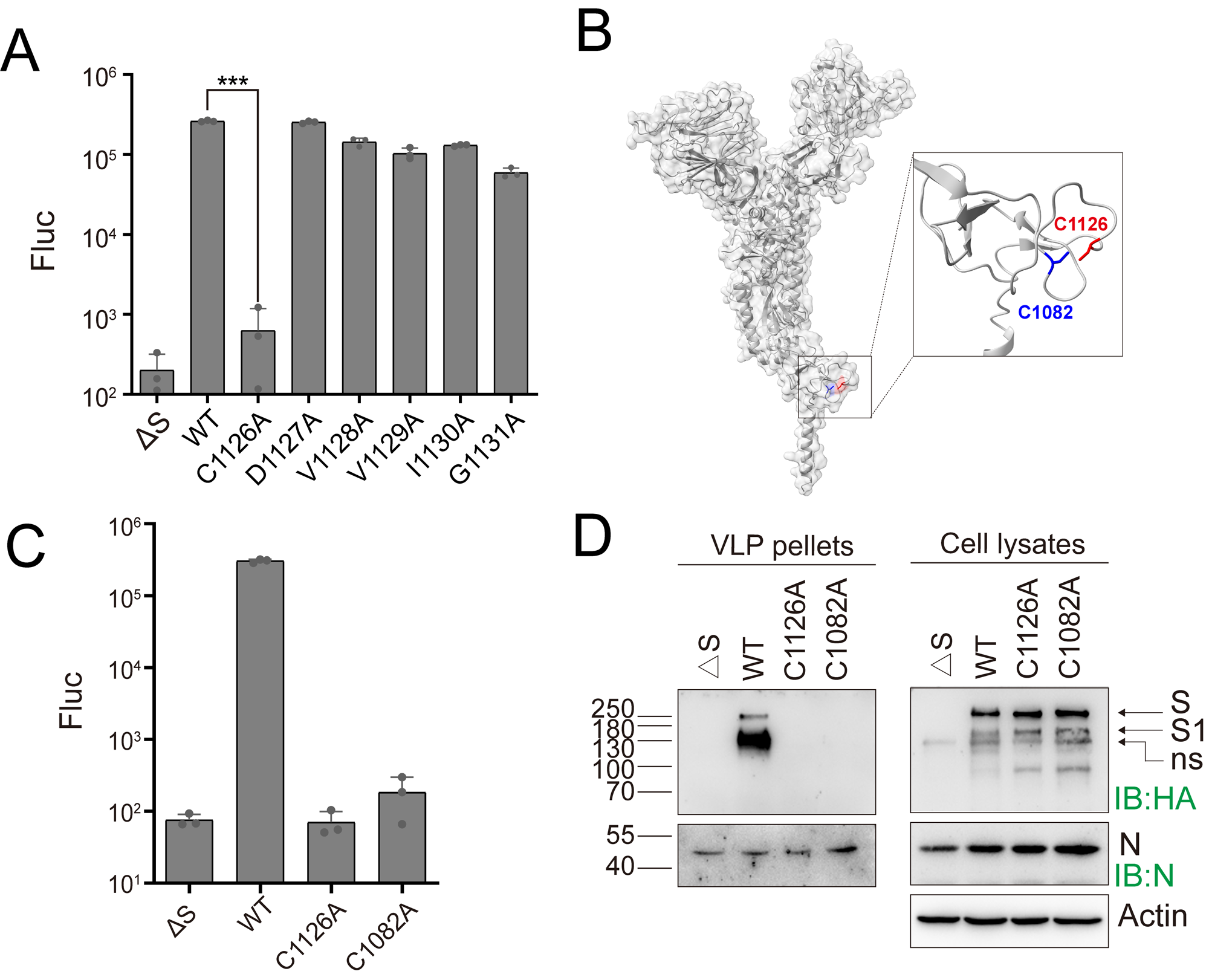

Supplement: S3 Fig — (A) The infectivity of SC2-VLP mutants. The packaged SC2-VLPs with single alanine mutations of the Mut2 and Mut3 were used to infect HEK293T-ACE2&TMPRSS2 cells, and intracellular Firefly luciferase activities were determined at 24 hours post-infection (mean values ± SDs, n = 3, ***P < 0.001; two-tailed, unpaired t-test). (B) Structural visualization of the residue C1126 in the prefusion Spike. The enlarged region (boxed) indicated the potential disulfide bond formed between C1126 (red) and C1082 (blue). (C) The infectivity of SC2-VLPs with the C1126A and C1082A mutants. The packaged SC2-VLPs-(Spike·C1126A) and SC2-VLPs-(Spike·C1082A) were used to infect HEK293T-ACE2&TMPRSS2 cells, and intracellular Firefly luciferase , i.e.,s were determined at 24 hours post-infection (mean values ± SDs, n = 3). (D) Western blotting of SC2-VLPs with the C1126A and C1082A mutants. The SC2-VLP-containing supernatants precipitated by methanol and the packaging cell lysates were subjected to Western blotting assay with the indicated antibodies (IB). The values to the left of the blots are molecular sizes in kilodaltons. The representative images shown for each group from multiple independent experiments. (TIF) [file ppat.1013526.s003.tif]

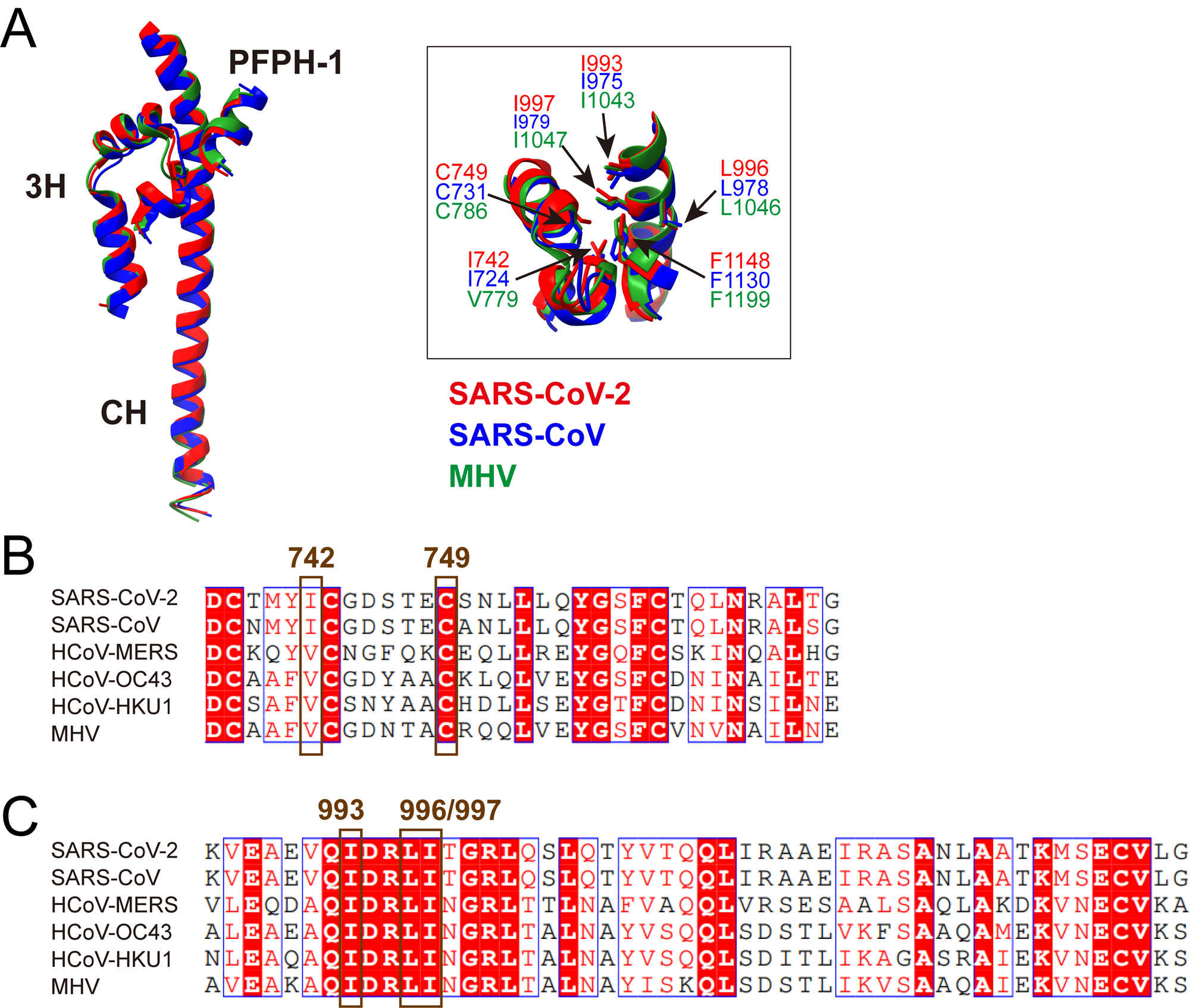

Supplement: S4 Fig — (A) The structural alignments of PFPH-1, 3H and CH across the betacoronaviruses. The image was generated by aligning PFPH-1, 3H, and CH domains of the SARS-CoV-2 (red, PDB 8FDW) with SARS-CoV (blue, PDB 6M3W) and MHV (green, PDB 6B3O), which yielded a root mean square deviations (RMSDs) of 0.804 Å (SARS-CoV-2 with SARS-CoV), 0.589 Å (SARS-CoV-2 with MHV) and 0.649 Å (SARS-CoV with MHV), respectively. The enlarged box indicated the key hydrophobic residues. (B-C) The amino acid sequence alignments of 3H (panel B) and CH (panel C) were performed using Clustal Omega and ESPript 3.0. Red highlighting represents 100% identity, whereas blue boxed red fonts shows a global score of 70% identity based on ESPript 3.0 parameters. Brown boxes indicate the key hydrophobic residues. (TIF) [file ppat.1013526.s004.tif]

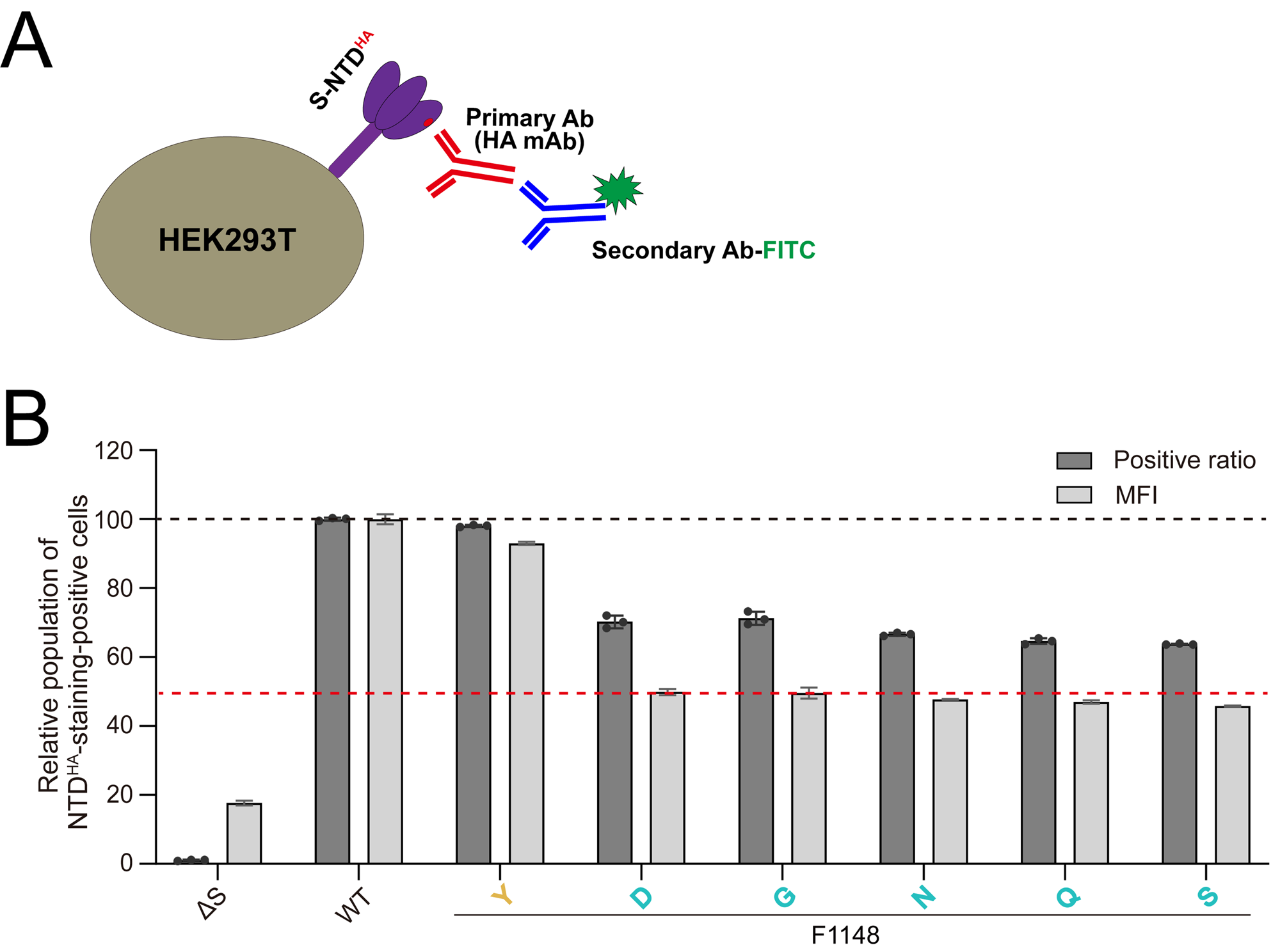

Supplement: S5 Fig — (A) Schematic representation experimental design of the cell surface staining of Spike-NTDHA. Spike-NTDHA was expressed on the surface of HEK293T cells, followed by incubation with primary antibody (Anti-HA mAb) and secondary antibody at 4°C for 1 hours. The percentage of FITC-positive cells and the mean fluorescence intensity (MFI) was determined by flow cytometry. (B) The population of NTDHA-staining-positive cells was determined by normalizing the percentage of FITC-positive cells and the mean fluorescence intensity (MFI) of the Spike.F1148 mutants to that of WT (mean values ± SDs, n = 3). (TIF) [file ppat.1013526.s005.tif]

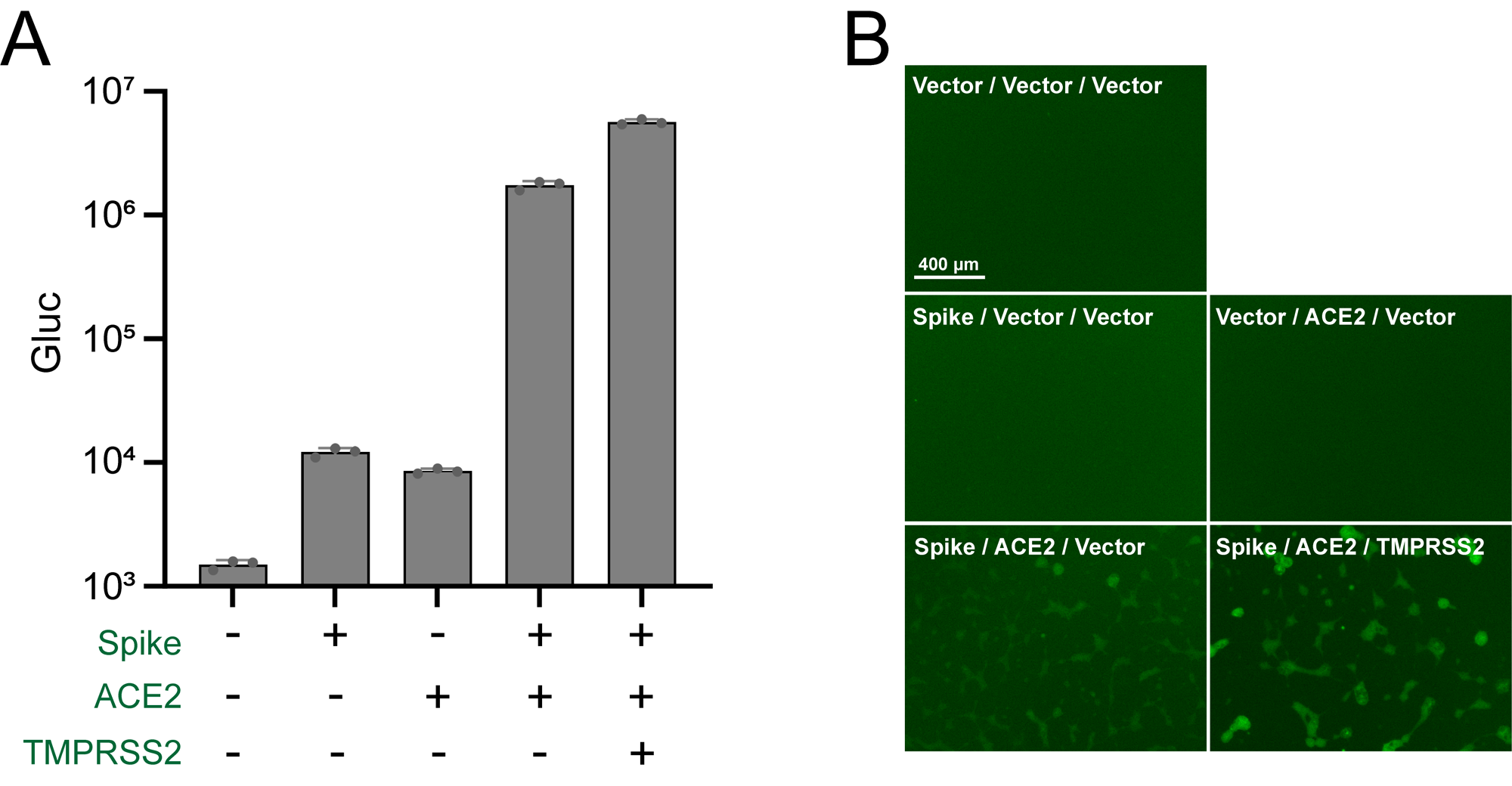

Supplement: S6 Fig — (A) Quantitative analysis of Spike-mediated membrane fusion efficiency. The donor cell population expressing Spike was incubated with different types of recipient cell populations at 37°C, and the intracellular Gluc luciferase activities were determined at 24 hours post-incubation. (mean values ± SDs, n = 3). (B) The fluorescence images of panel A. Incubate the donor cell population expressing Spike with the recipient cell population expressing ACE2 at 37°C, fluorescence images were acquired at 24 hours post-incubation. (TIF) [file ppat.1013526.s006.tif]

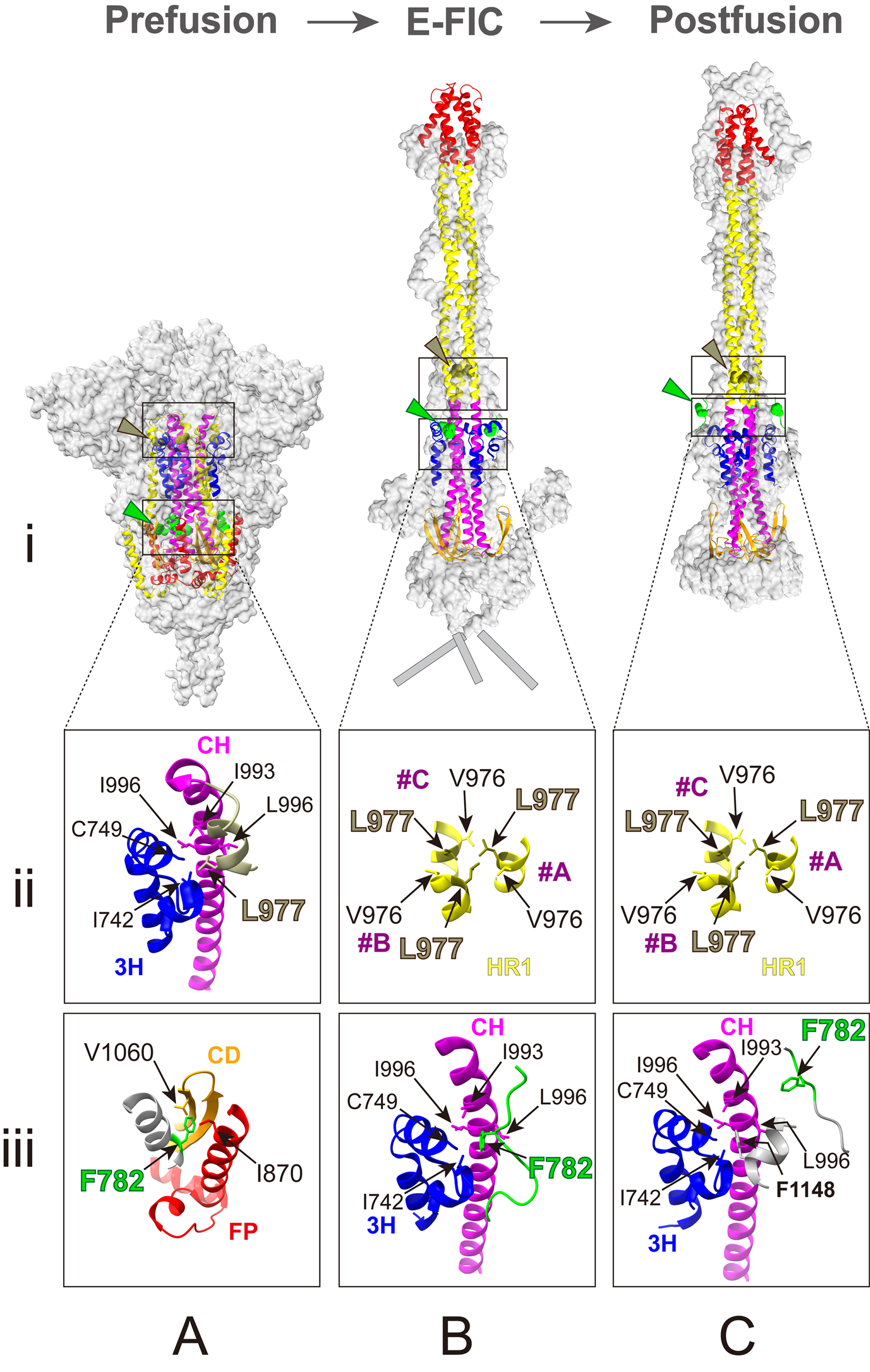

Supplement: S7 Fig — (A-C) Schematic of the “hydrolock interaction” model. The images were generated using PDB 6XR8, PDB 8Z7P and PDB 8FDW, illustrating the overview (i), L977 (ii) and F782 (iii) at different conformational stages (A, prefusion; B, E-FIC, early fusion intermediate conformation. C, postfusion). The 3H (blue), CH (magenta), HR1 (yellow), FP (red), CD (orange), helix976–984 (khaki) and loop776–785 (green) are shown. The khaki and green triangular arrows indicate the residues L977 and F782, respectively. (TIF) [file ppat.1013526.s007.tif]

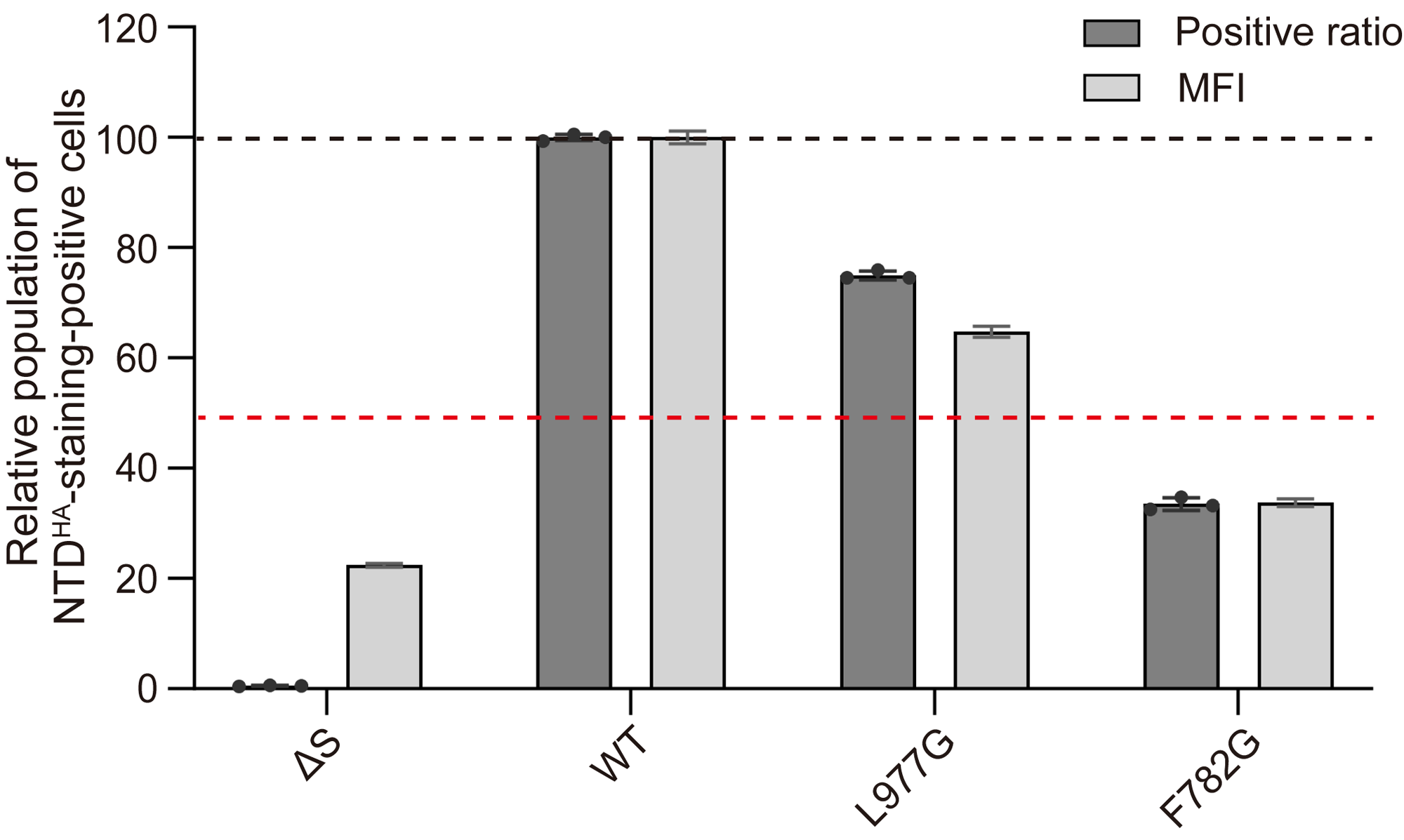

Supplement: S8 Fig — The population of NTDHA-staining-positive cells was determined by normalizing the percentage of FITC-positive cells and the mean fluorescence intensity (MFI) of the Spike mutants to that of WT (mean values ± SDs, n = 3). (TIF) [file ppat.1013526.s008.tif]

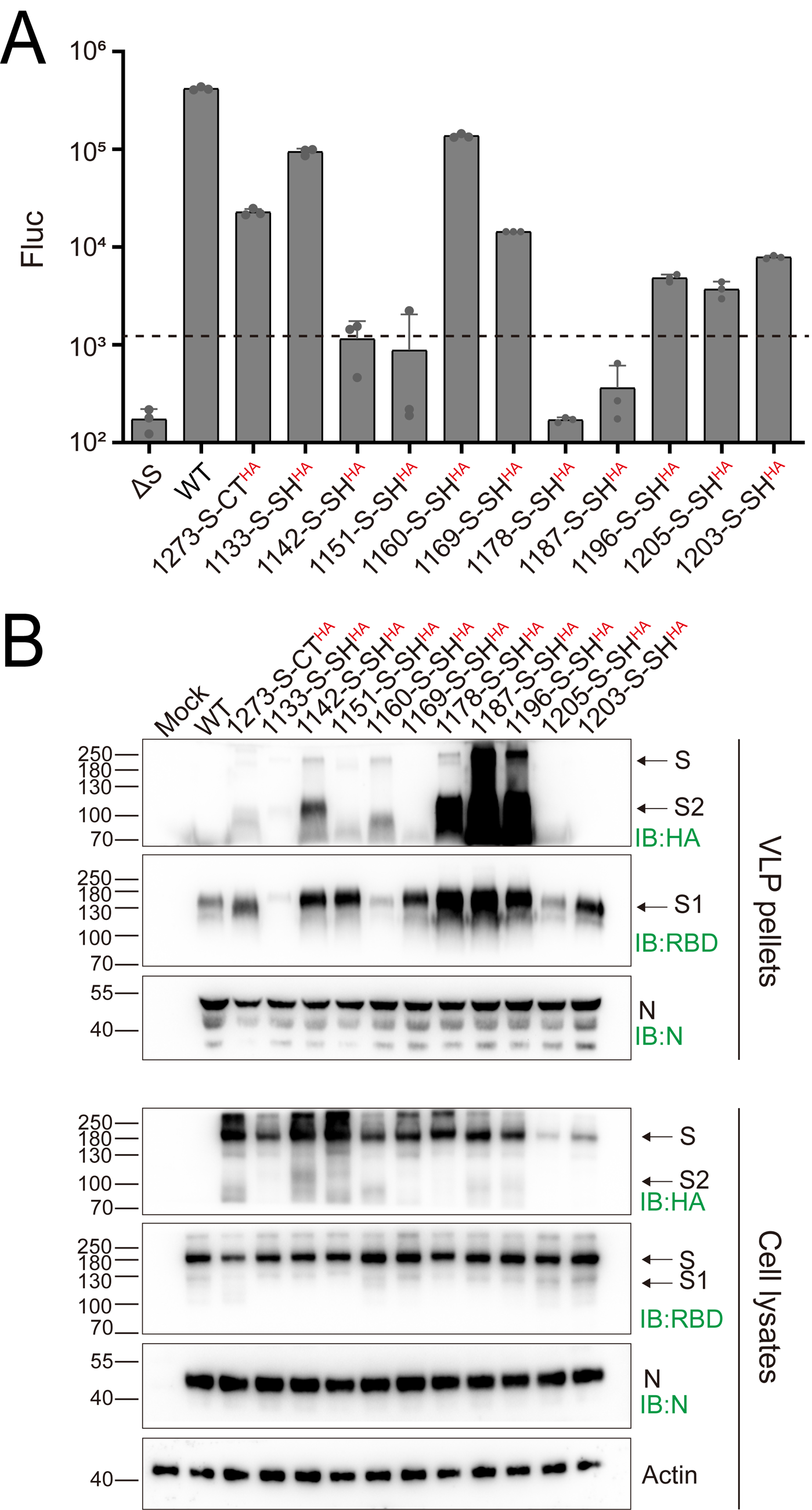

Supplement: S9 Fig — (A) The infectivity of SC2-VLP with SpikeHA linear epitope replacement mutants. The packaged SC2-VLPs-(S-SHHA) were used to infected HEK293T-ACE2&TMPRSS2 cells, and intracellular Firefly luciferase activities were determined at 24 hours post-infection (mean values ± SDs, n = 3). The black dashed line represents the baseline for detectable infection signals, which is applicable for subsequent neutralization assays. (B) Western blotting of SC2-VLPs-(S-SHHA). The SC2-VLP-containing supernatants precipitated by methanol and the packaging cell lysates were subjected to Western blotting assay with the indicated antibodies (IB). The values to the left of the blots are molecular sizes in kilodaltons. Mock, untransfected HEK293T control. (TIF) [file ppat.1013526.s009.tif]

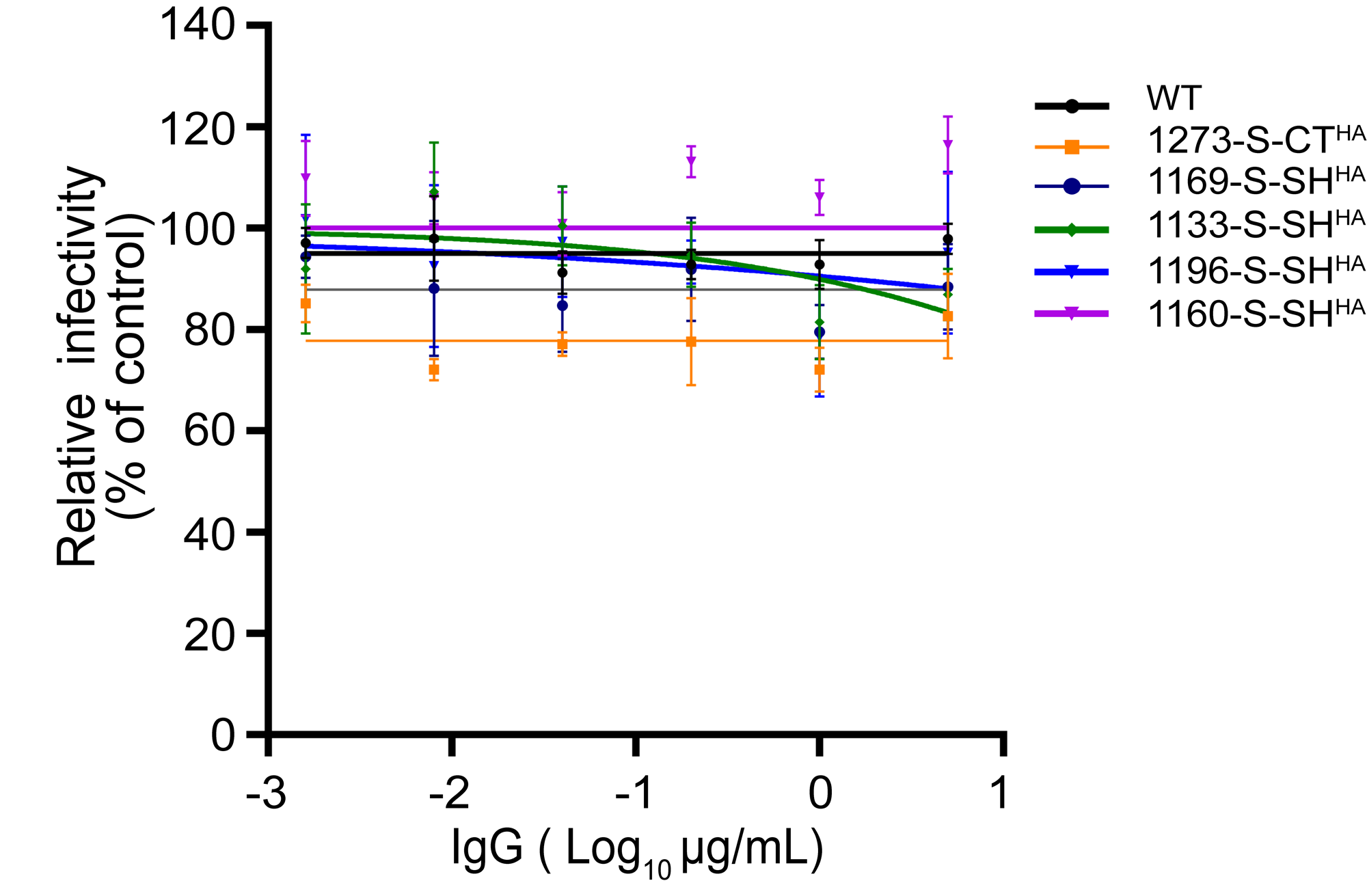

Supplement: S10 Fig — The infectivity of SC2-VLP-(S-SHHA) after incubation with a 5-fold concentration gradient of rabbit IgG at 37°C for 1 hour. The intracellular Firefly luciferase activities were determined at 24 hours post-infection. The relative infectivity was determined by normalizing the luciferase activity of SC2-VLPs incubated with IgG to that of SC2-VLPs incubated with medium as a control. The curve fitting was performed using GraphPad Prism software. (mean values ± SDs, n = 3). (TIF) [file ppat.1013526.s010.tif]

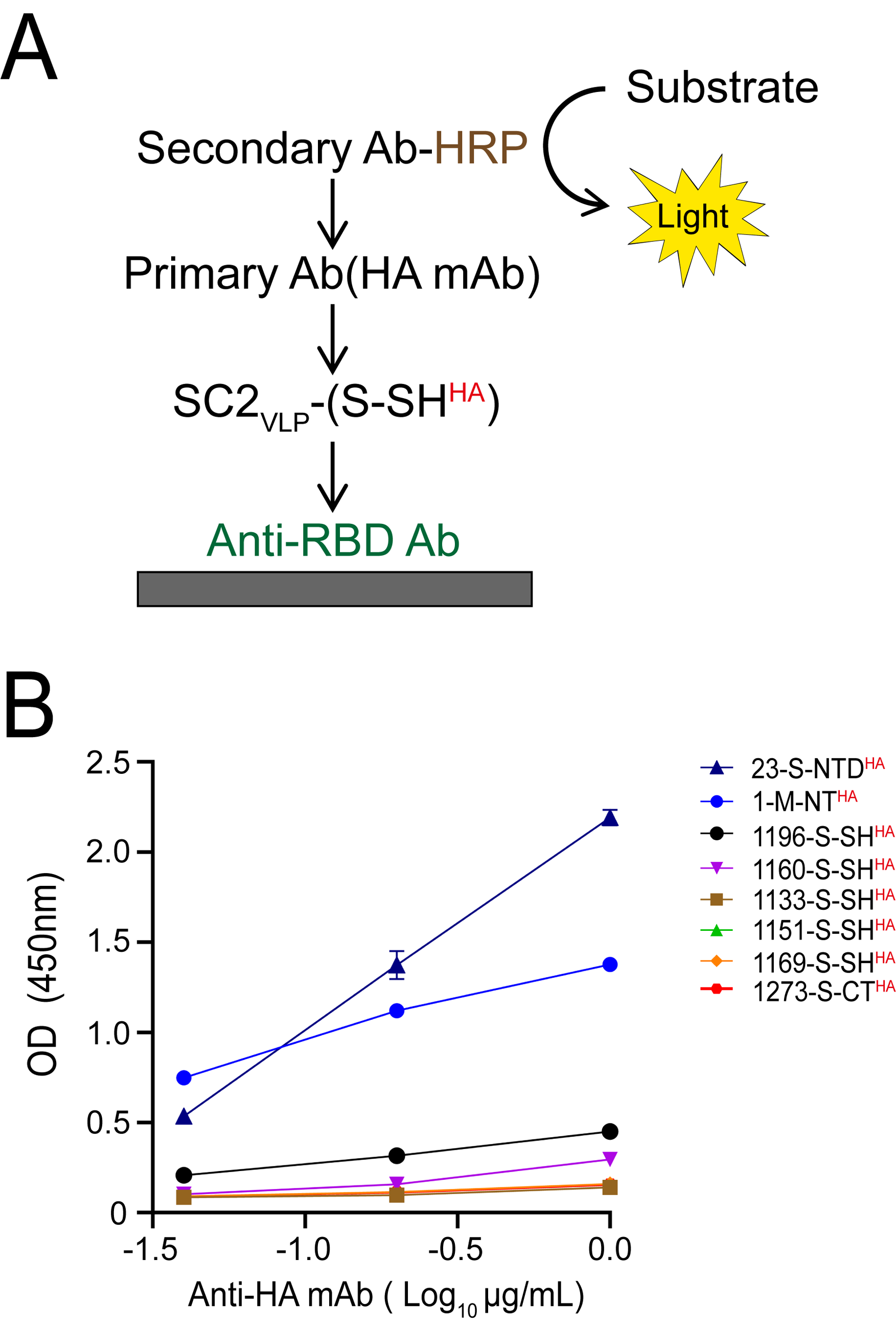

Supplement: S11 Fig — (A) Experimental schematic for assessing SH region antibody accessibility using ELISA. Anti-Spike RBD mAb-coated 96-well plates were used to capture SC2-VLPs-(S-SHHA). Sequential incubations were performed by adding the primary antibody (anti-HA mAb), followed by an HRP-conjugated secondary antibody. A chemiluminescent substrate was added to detect the signal. (B) Quantitative analysis of SH accessibility using OD450 measurements. A 5-fold concentration gradient of anti-HA mAb was applied, and the OD450 values were measured to reflect the accessibility of different regions of SH. (mean values ± SDs, n = 3). (TIF) [file ppat.1013526.s011.tif]

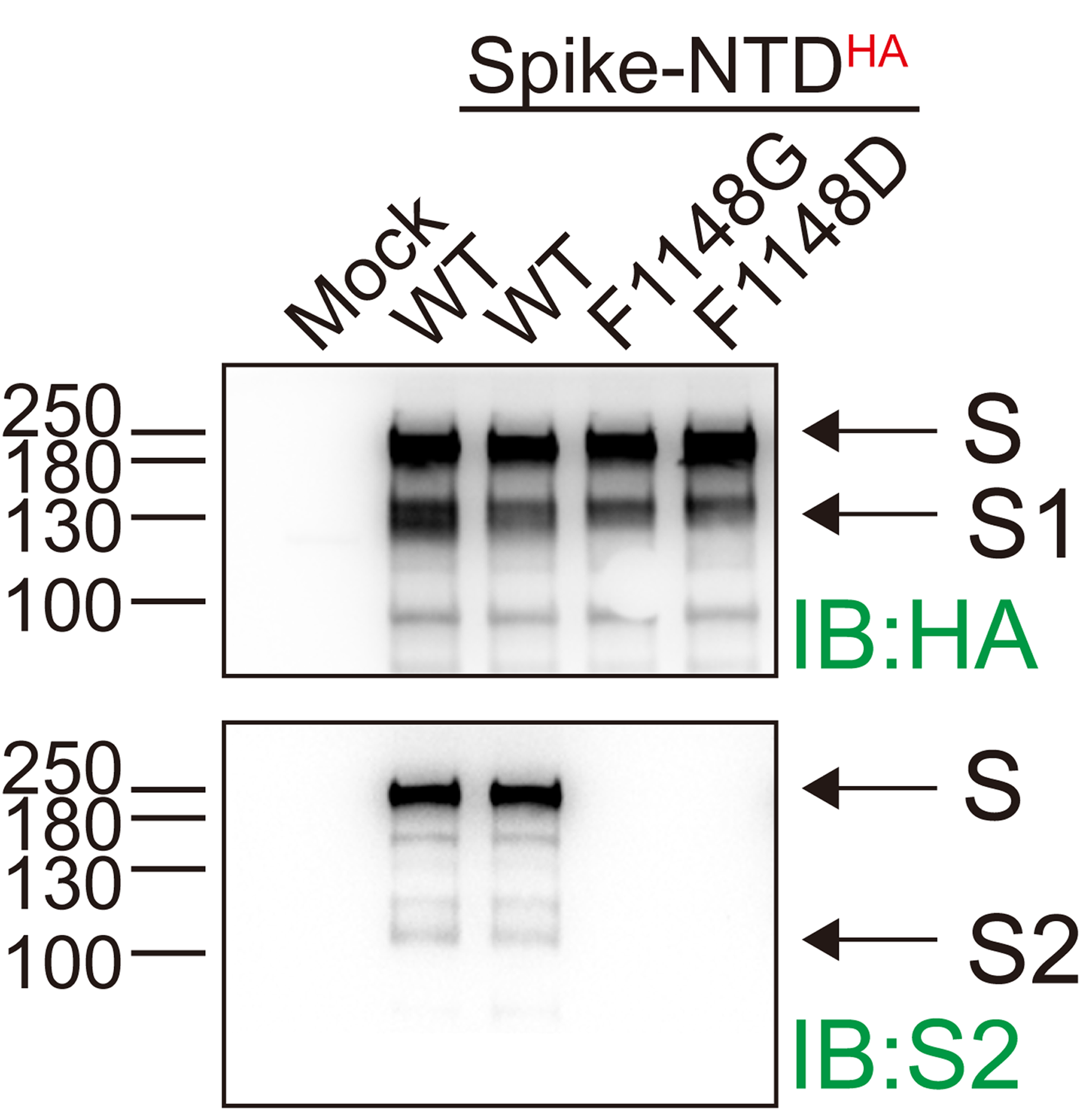

Supplement: S12 Fig — F1148 mutants. The plasmids Spike·WT and Spike·F1148 mutants were transfected into HEK293T cells. After 24 hours, cell lysates were harvested and subjected to Western blotting assay with the indicated antibodies (IB). The values to the left of the blots are molecular sizes in kilodaltons. Mock: naive HEK293T cells without plasmid transfection. (TIF) [file ppat.1013526.s012.tif]
