# Supplementary material for: The F1148 hydrophobic lock: A critical determinant of SARS-CoV-2 spike protein-mediated membrane fusion via the 3H/CH cavity
Source: PLoS Pathog. 2025 Sep 19;21(9):e1013526. doi: 10.1371/journal.ppat.1013526 (PMC12459827; doi:10.1371/journal.ppat.1013526)
Supplement: S1 Text — (DOC) [file ppat.1013526.s017.doc]

**>ACE2-GFP-FC**

MDPKGLLSLTFVLFLSLAFGTIEEQAKTFLDKFNHEAEDLFYQSSLASWNYNTNITEENVQNMNNAGDKWSAFLKEQSTLAQMYPLQEIQNLTVKLQLQALQQNGSSVLSEDKSKRLNTILNTMSTIYSTGKVCNPDNPQECLLLEPGLNEIMANSLDYNERLWAWESWRSEVGKQLRPLYEEYVVLKNEMARANHYEDYGDYWRGDYEVNGVDGYDYSRGQLIEDVEHTFEEIKPLYEHLHAYVRAKLMNAYPSYISPIGCLPAHLLGDMWGRFWTNLYSLTVPFGQKPNIDVTDAMVDQAWDAQRIFKEAEKFFVSVGLPNMTQGFWENSMLTDPGNVQKAVCHPTAWDLGKGDFRILMCTKVTMDDFLTAHHEMGHIQYDMAYAAQPFLLRNGANEGFHEAVGEIMSLSAATPKHLKSIGLLSPDFQEDNETEINFLLKQALTIVGTLPFTYMLEKWRWMVFKGEIPKDQWMKKWWEMKREIVGVVEPVPHDETYCDPASLFHVSNDYSFIRYYTRTLYQFQFQEALCQAAKHEGPLHKCDISNSTEAGQKLFNMLRLGKSEPWTLALENVVGAKNMNVRPLLNYFEPLFTWLKDQNKNSFVGWSTDWSPYADQSIKVRISLKSALGDKAYEWNDNEMYLFRSSVAYAMRQYFLKVKNQMILFGEEDVRVANLKPRISFNFFVTAPKNVSDIIPRTEVEKAIRMSRSRINDAFRLNDNSLEFLGIQPTLGPPNQPPVSGSSSNSSKGEELFTGVVPILVELDGDVNGHKFSVRGEGEGDATIGKLTLKFICTTGKLPVPWPTLVTTLTYGVQCFSRYPDHMKRHDFFKSAMPEGYVQERTISFKDDGKYKTRAVVKFEGDTLVNRIELKGTDFKEDGNILGHKLEYNFNSHNVYITADKQKNGIKANFTVRHNVEDGSVQLADHYQQNTPIGDGPVLLPDNHYLSTQTVLSKDPNEKRDHMVLHEYVNAAGITGTDKTHTCPPCPAPELLGGPSVFLFPPKPKDTLMISRTPEVTCVVVDVSHEDPEVKFNWYVDGVEVHNAKTKPREEQYNSTYRVVSVLTVLHQDWLNGKEYKCKVSNKALPAPIEKTISKAKGQPREPQVYTLPPSRDELTKNQVSLTCLVKGFYPSDIAVEWESNGQPENNYKTTPPVLDSDGSFFLYSKLTVDKSRWQQGNVFSCSVMHEALHNHYTQKSLSLSPGKGGHHHHHH*

(ATGGATCCCAAGGGGCTCCTCTCCTTGACCTTCGTGCTGTTTCTCTCCCTGGCTTTTGGGACCATTGAGGAACAGGCCAAGACATTTTTGGACAAGTTTAACCACGAAGCCGAAGACCTGTTCTATCAAAGTTCACTTGCTTCTTGGAATTATAACACCAATATTACTGAAGAGAATGTCCAAAACATGAATAATGCTGGGGACAAATGGTCTGCCTTTTTAAAGGAACAGTCCACACTTGCCCAAATGTATCCACTACAAGAAATTCAGAATCTCACAGTCAAGCTTCAGCTGCAGGCTCTTCAGCAAAATGGGTCTTCAGTGCTCTCAGAAGACAAGAGCAAACGGTTGAACACAATTCTAAATACAATGAGCACCATCTACAGTACTGGAAAAGTTTGTAACCCAGATAATCCACAAGAATGCTTATTACTTGAACCAGGTTTGAATGAAATAATGGCAAACAGTTTAGACTACAATGAGAGGCTCTGGGCTTGGGAAAGCTGGAGATCTGAGGTCGGCAAGCAGCTGAGGCCATTATATGAAGAGTATGTGGTCTTGAAAAATGAGATGGCAAGAGCAAATCATTATGAGGACTATGGGGATTATTGGAGAGGAGACTATGAAGTAAATGGGGTAGATGGCTATGACTACAGCCGCGGCCAGTTGATTGAAGATGTGGAACATACCTTTGAAGAGATTAAACCATTATATGAACATCTTCATGCCTATGTGAGGGCAAAGTTGATGAATGCCTATCCTTCCTATATCAGTCCAATTGGATGCCTCCCTGCTCATTTGCTTGGTGATATGTGGGGTAGATTTTGGACAAATCTGTACTCTTTGACAGTTCCCTTTGGACAGAAACCAAACATAGATGTTACTGATGCAATGGTGGACCAGGCCTGGGATGCACAGAGAATATTCAAGGAGGCCGAGAAGTTCTTTGTATCTGTTGGTCTTCCTAATATGACTCAAGGATTCTGGGAAAATTCCATGCTAACGGACCCAGGAAATGTTCAGAAAGCAGTCTGCCATCCCACAGCTTGGGACCTGGGGAAGGGCGACTTCAGGATCCTTATGTGCACAAAGGTGACAATGGACGACTTCCTGACAGCTCATCATGAGATGGGGCATATCCAGTATGATATGGCATATGCTGCACAACCTTTTCTGCTAAGAAATGGAGCTAATGAAGGATTCCATGAAGCTGTTGGGGAAATCATGTCACTTTCTGCAGCCACACCTAAGCATTTAAAATCCATTGGTCTTCTGTCACCCGATTTTCAAGAAGACAATGAAACAGAAATAAACTTCCTGCTCAAACAAGCACTCACGATTGTTGGGACTCTGCCATTTACTTACATGTTAGAGAAGTGGAGGTGGATGGTCTTTAAAGGGGAAATTCCCAAAGACCAGTGGATGAAAAAGTGGTGGGAGATGAAGCGAGAGATAGTTGGGGTGGTGGAACCTGTGCCCCATGATGAAACATACTGTGACCCCGCATCTCTGTTCCATGTTTCTAATGATTACTCATTCATTCGATATTACACAAGGACCCTTTACCAATTCCAGTTTCAAGAAGCACTTTGTCAAGCAGCTAAACATGAAGGCCCTCTGCACAAATGTGACATCTCAAACTCTACAGAAGCTGGACAGAAACTGTTCAATATGCTGAGGCTTGGAAAATCAGAACCCTGGACCCTAGCATTGGAAAATGTTGTAGGAGCAAAGAACATGAATGTAAGGCCACTGCTCAACTACTTTGAGCCCTTATTTACCTGGCTGAAAGACCAGAACAAGAATTCTTTTGTGGGATGGAGTACCGACTGGAGTCCATATGCAGACCAAAGCATCAAAGTGAGGATAAGCCTAAAATCAGCTCTTGGAGATAAAGCATATGAATGGAACGACAATGAAATGTACCTGTTCCGATCATCTGTTGCATATGCTATGAGGCAGTACTTTTTAAAAGTAAAAAATCAGATGATTCTTTTTGGGGAGGAGGATGTGCGAGTGGCTAATTTGAAACCAAGAATCTCCTTTAATTTCTTTGTCACTGCACCTAAAAATGTGTCTGATATCATTCCTAGAACTGAAGTTGAAAAGGCCATCAGGATGTCCCGGAGCCGTATCAATGATGCTTTCCGTCTGAATGACAACAGCCTAGAGTTTCTGGGGATACAGCCAACACTTGGACCTCCTAACCAGCCCCCTGTTTCCGGCAGCAGCTCGAATTCTTCCAAAGGAGAAGAACTGTTTACCGGTGTTGTGCCAATTTTGGTTGAACTCGATGGTGATGTCAACGGACATAAGTTCTCAGTGAGAGGCGAAGGAGAAGGTGACGCCACCATTGGAAAATTGACTCTTAAATTCATCTGTACTACTGGTAAACTTCCTGTACCATGGCCGACTCTCGTAACAACGCTTACGTACGGAGTTCAGTGCTTTTCGAGATACCCAGACCATATGAAAAGACATGACTTTTTTAAGTCGGCTATGCCTGAAGGTTACGTGCAAGAAAGAACAATTTCGTTCAAAGATGATGGAAAATATAAAACTAGAGCAGTTGTTAAATTTGAAGGAGATACTTTGGTTAACCGCATTGAACTGAAAGGAACAGATTTTAAAGAAGATGGTAATATTCTTGGACACAAACTCGAATACAATTTTAATAGTCATAACGTATACATCACTGCTGATAAGCAAAAGAACGGAATTAAAGCGAATTTCACAGTACGCCATAATGTAGAAGATGGCAGTGTTCAACTTGCCGACCATTACCAACAAAACACCCCTATTGGAGACGGTCCGGTACTTCTTCCTGATAATCACTACCTCTCAACACAAACAGTCCTGAGCAAAGATCCAAATGAAAAACGTGACCACATGGTCCTTCATGAGTATGTAAATGCTGCTGGGATTACAGGTACCGACAAAACTCACACATGCCCACCGTGCCCAGCACCTGAACTCCTGGGGGGACCGTCAGTCTTCCTCTTCCCCCCAAAACCCAAGGACACCCTCATGATCTCCCGGACCCCTGAGGTCACATGCGTGGTGGTGGACGTGAGCCACGAAGACCCTGAGGTCAAGTTCAACTGGTACGTGGACGGCGTGGAGGTGCATAATGCCAAGACAAAGCCGCGGGAGGAGCAGTACAACAGCACGTACCGTGTGGTCAGCGTCCTCACCGTCCTGCACCAGGACTGGCTGAATGGCAAGGAGTACAAGTGCAAGGTCTCCAACAAAGCCCTCCCAGCCCCCATCGAGAAAACCATCTCCAAAGCCAAAGGGCAGCCCCGAGAACCACAGGTGTATACCCTGCCCCCATCCCGGGATGAGCTGACCAAGAACCAGGTCAGCCTGACCTGCCTGGTCAAAGGCTTCTATCCCAGCGACATCGCCGTGGAGTGGGAGAGCAATGGGCAGCCGGAGAACAACTACAAGACCACGCCTCCCGTGCTGGACTCCGACGGCTCCTTCTTCCTCTACAGCAAGCTCACCGTGGACAAGAGCAGGTGGCAGCAGGGGAACGTCTTCTCATGCTCCGTGATGCATGAGGCTCTGCACAACCACTACACGCAGAAGAGCCTCTCCCTGTCTCCGGGTAAAGGCGGCCATCATCACCATCACCATTAA)

**>VNnGluc-cfaN**

MVSKGEELFTGVVPILVELDGDVNGHKFSVSGEGEGDATYGKLTLKLICTTGKLPVPWPTLVTTLGYGLQCFARYPDHMKQHDFFKSAMPEGYVQERTIFFKDDGNYKTRAEVKFEGDTLVNRIELKGIDFKEDGNILGHKLEYNYNSHNVYITADKQKNGIKANFKIRHNIESAAKPTENNEDFNIVAVASNFATTDLDADRGKLPGKKLPLEVLKEMEANARKAGCTRGCLICLSHIKCTPKMKKFIPGRCHTYEGDKESAQGGIGLECLSYDTEILTVEYGFLPIGKIVEERIECTVYTVDKNGFVYTQPIAQWHNRGEQEVFEYCLEDGSIIRATKDHKFMTTDGQMLPIDEIFERGLDLKQVDGLPGS*

(ATGGTGAGCAAGGGCGAGGAGCTGTTCACCGGGGTGGTGCCCATCCTGGTCGAGCTGGACGGCGACGTAAACGGCCACAAGTTCAGCGTGTCCGGCGAGGGCGAGGGCGATGCCACCTACGGCAAGCTGACCCTGAAGCTGATCTGCACCACCGGCAAGCTGCCCGTGCCCTGGCCCACCCTGGTGACCACCCTGGGCTACGGCCTGCAGTGCTTCGCCCGCTACCCCGACCACATGAAGCAGCACGACTTCTTCAAGTCCGCCATGCCCGAAGGCTACGTCCAGGAGCGCACCATCTTCTTCAAGGACGACGGCAACTACAAGACCCGCGCCGAGGTGAAGTTCGAGGGCGACACCCTGGTGAACCGCATCGAGCTGAAGGGCATCGACTTCAAGGAGGACGGCAACATCCTGGGGCACAAGCTGGAGTACAACTACAACAGCCACAACGTCTATATCACCGCCGACAAGCAGAAGAACGGCATCAAGGCCAACTTCAAGATCCGCCACAACATCGAGAGCGCTGCCAAGCCCACCGAGAACAACGAAGACTTCAACATCGTGGCCGTGGCCAGCAACTTCGCGACCACGGATCTCGATGCTGACCGCGGGAAGTTGCCCGGCAAGAAGCTGCCGCTGGAGGTGCTCAAAGAGATGGAAGCCAATGCCCGGAAAGCTGGCTGCACCAGGGGCTGTCTGATCTGCCTGTCCCACATCAAGTGCACGCCCAAGATGAAGAAGTTCATCCCAGGACGCTGCCACACCTACGAAGGCGACAAAGAGTCCGCACAGGGCGGCATAGGCCTCGAGTGCCTGAGCTACGACACCGAGATCCTGACCGTGGAGTACGGCTTCCTGCCCATCGGCAAGATCGTGGAGGAGCGCATCGAGTGCACCGTGTACACCGTGGACAAGAACGGCTTCGTGTACACCCAGCCCATCGCCCAGTGGCACAACCGCGGCGAGCAGGAGGTGTTCGAGTACTGCCTGGAGGACGGCAGCATCATCCGCGCCACCAAGGACCACAAGTTCATGACCACCGACGGCCAGATGCTGCCCATCGACGAGATCTTCGAGCGCGGCCTGGACCTGAAGCAGGTGGACGGCCTGCCCGGATCCTAA)

**>CfaC-cGlucVC**

MVKIISRKSLGTQNVYDIGVEKDHNFLLKNGLVASNCTEAIVDIPEIPGFKDLEPMEQFIAQVDLCVDCTTGCLKGLANVQCSDLLKKWLPQRCATFASKIQGQVDKIKGAGGDGGGGSGGGGSGGGGSGGGGSRDHMVSADGGVQLADHYQQNTPIGDGPVLLPDNHYLSYQSALSKDPNEKRDHMVLLEFVTAAGITLGMDELYK*

(ATGGTGAAGATCATCAGCCGCAAGAGCCTGGGCACCCAGAACGTGTACGACATCGGCGTGGAGAAGGACCACAACTTCCTGCTGAAGAACGGCCTGGTGGCCAGCAACTGTACAGAGGCGATCGTCGACATTCCTGAGATTCCTGGGTTCAAGGACTTGGAGCCCATGGAGCAGTTCATCGCACAGGTCGATCTGTGTGTGGACTGCACAACTGGCTGCCTCAAAGGGCTTGCCAACGTGCAGTGTTCTGACCTGCTCAAGAAGTGGCTGCCGCAACGCTGTGCGACCTTTGCCAGCAAGATCCAGGGCCAGGTGGACAAGATCAAGGGGGCCGGTGGTGACGGCGGCGGCGGCAGCGGCGGCGGCGGCAGCGGCGGCGGCGGCAGCGGCGGCGGCGGCAGCCGTGACCACATGGTCAGCGCTGACGGCGGCGTGCAGCTCGCCGACCACTACCAGCAGAACACCCCCATCGGCGACGGCCCCGTGCTGCTGCCCGACAACCACTACCTGAGCTACCAGTCCGCCCTGAGCAAAGACCCCAACGAGAAGCGCGATCACATGGTCCTGCTGGAGTTCGTGACCGCCGCCGGGATCACTCTCGGCATGGACGAGCTGTACAAGTAA)
