# Supplementary material for: The F1148 hydrophobic lock: A critical determinant of SARS-CoV-2 spike protein-mediated membrane fusion via the 3H/CH cavity
Source: PLoS Pathog. 2025 Sep 19;21(9):e1013526. doi: 10.1371/journal.ppat.1013526 (PMC12459827; doi:10.1371/journal.ppat.1013526)
Supplement: Attachment — Submitted filename: Response to Reviewers.doc [file ppat.1013526.s019.doc]

**July 28, 2025**

**Professor Tongqing Zhou**

**Guest editor, PloS Pathogens**

We are grateful for you and the reviewers for taking your time in reviewing our paper “The F1148 Hydrophobic Lock: A Critical Determinant of SARS-CoV-2 Spike Protein-Mediated Membrane Fusion via the 3H/CH Cavity” (PPATHOGENS-D-25-01115).

In response to the reviewers’ comments and suggestions, we have designed and conducted several additional experiments to strengthen our findings. 1) We quantitatively assessed the cell surface staining of Spike-NTD^HA^ variants (with the HA tag inserted after Q23) by flow cytometry using anti-HA antibodies. 2) We repeated the cell–cell membrane fusion assays and integrated the new results with the original dataset. 3) We evaluated the effect of trypsin treatment on Spike cell surface expression to assess its suitability for use in flow cytometry experiments. 4) We conducted additional experiments using a concentration gradient of XG005 on wild-type Spike to examine the correlation between inhibition of membrane fusion and reduced ACE2 binding.

In addition to the experimental updates, we have enhanced data analysis and presentation with the following improvements: inclusion of Mean Fluorescence Intensity (MFI) quantification in flow cytometry results; standardization of color schemes in structural illustrations; uniform Y-axis labeling across relevant bar graphs for improved clarity. We have also thoroughly revised the manuscript for language and clarity.

Below, we provide detailed responses to each reviewer’s comment, with our responses indicated in blue. We respectfully ask whether, in its current form, the manuscript is now suitable for publication in PLOS Pathogens.

**Part I - Summary**

Reviewer #1: This manuscript titled “The F1148 Hydrophobic Lock: A Critical Determinant of SARS-CoV-2 Spike Protein-Mediated Membrane Fusion via the 3H/CH Cavity” presents a systematic investigation of the hydrophobic interaction centered on residue F1148 in the spike stem helix (SH) region. For clss-I viral fusion proteins, the HR1/HR2 6-helix bundle is the core structure of membrane fusion, while the critical domains or residues that drive this process are still unclear. In this study, the authors characterize a “hydrolock” interaction formed by F1148 and the 3H/CH cavity as an essential structural and functional element for spike-mediated membrane fusion. Overall, this study is novel and interesting. However, there are some concerns in this study.

We sincerely appreciate the reviewer #1’s thoughtful evaluation of our manuscript and his/her recognition of the novelty in characterizing the F1148 “hydrolock” mechanism. We agree with the reviewer that further clarification is needed regarding certain aspects of the study.

Reviewer #2: In this work, Lei and colleagues performed systematic mutagenesis within a conserved region of SARS-CoV-2 spike protein, the stem-helix (SH) on S2 subunit, to identify key residues involved in viral infection and membrane fusion. The results highlighted helices preserved in the postfusion spike, which the authors termed as postfusion-preserved helices (PFPH1, PFPH2 and PFPH3). Through structural analyses of the spike protein in prefusion, early-fusion intermediate and postfusion states, the authors identified a hydrophobic pocket – referred to as the “hydrolock” -- which accommodates hydrophobic residues L977, F782 and F1148 in a state-dependent manner. This pocket interacts with these residues alternately in prefusion, early-fusion intermediate and postfusion states. Mutating these residues to hydrophilic amino acids or disrupting the hydrophobic pocket significantly impaired spike-mediated viral infectivity. Moreover, neutralizing antibodies targeting the stem-helix region were found to bind the spike and clash with its postfusion state. To further dissect the neutralizing mechanism of these antibodies, the authors employed HA replacement mutagenesis to map critical regions within the stem-helix domain. They found that antibody binding to regions adjacent to PFPHs is essential for blocking membrane fusion. In summary, the study provides a detailed structural comparison of available spike models at different stages during fusion and identifies a conserved hydrophobic cavity that is critical for infectivity, likely through facilitating S2 refolding. Given the conservation of this region across coronaviruses, it represents a promising target for the development of broad-spectrum antiviral therapies. This work is well-performed and incorporates multiple assays – including infectivity, ACE2 binding, membrane-membrane fusion and epitope exposure for neutralization -- to functionally characterize spike mutants. The systematic mutagenesis of the stem-helix region provides valuable insights for pan-coronavirus vaccine and therapeutic development.

Thank you for highlighting the strengths of our work and your positive reception of the key findings regarding the conserved hydrolock pocket. We greatly appreciate your insightful comments and agreement on the potential of this stem-helix region as a broad-spectrum antiviral target. Your constructive feedback has been valuable for strengthening our manuscript.

Reviewer #3: In this paper, the authors find that the hydrophobic interaction between F782/FF148 and 3H/CH cavity and also the postfusion-preserved helice-1 (PFPH-1)/PFPH-2 are important for conformational change of coronavirus spike protein and virus entry. Based on this research, they hypothesize a novel model of coronavirus entry. Overall, this is a good paper which can provide us with some new targets for antiviral drug and coronavirus vaccine design. However, there are some questions remaining to be addressed, please see the major and minor issues.

We sincerely appreciate Reviewer #3’s positive evaluation of our work and their recognition of its potential implications for antiviral drug and vaccine development. We are grateful for the constructive questions raised regarding both major and minor issues, which have helped us strengthen the manuscript.  Below we provide point-by-point responses to all concerns, with additional experimental data and clarifications incorporated into the revised version.

**Part II – Major Issues: Key Experiments Required for Acceptance**

**Reviewer #1:**

1. The F1148 hydrophilic mutants nearly abolished the virus entry (Fig. 3D) and severely impaired the membrane fusion efficiency (Fig. 3J) but still maintained more than 50% of ACE2-binding efficiency (Fig. 3H). The ACE2-binding ability is retained, which only means the RBD conformation can be maintained to some extent, but the overall prefusion conformation may still be changed, which could be the reason for the ability abolishment of virus entry and membrane fusion. A panel of antibodies targeting different regions of spike protein are needed to confirm the prefusion conformation like using flow cytometry.

We sincerely appreciate Reviewer #1’s insightful suggestion regarding the validation of the prefusion conformation. While we fully acknowledge the importance of employing conformation-sensitive antibodies targeting multiple regions of the Spike protein, we faced the following technical limitations: (1) anti-S2 antibodies exhibited poor accessibility to prefusion Spike proteins, as evidenced by the observation that anti-HA antibodies also failed to efficiently recognize the Spike protein in VLP particles when the HA linear epitope was replaced with a stem helix (Revised S11B Fig); and (2) the availability of reported antibodies recognizing conformational epitopes remains limited.

In response to the reviewer’s recommendation, we designed and performed an alternative experiment, now presented in the revised S5 Fig, in which we the cell-surface-expressed Spike-NTD^HA^ variants (HA tag inserted after Q23) was immunostained using anti-HA antibodies and subsequently analyzed by flow cytometry. The analysis showed that all hydrophilic mutations (F1148D, F1148G, F1148N, F1148Q, and F1148S) displayed reduced yet substantial anti-HA reactivity, with positive staining ratios exceeding 60% and mean fluorescence intensity (MFI) values above 45% compared to the wild-type (WT) control. This attenuated cell surface staining is likely attributable to conformational alterations in the Spike protein, which may also account for their reduced ACE2-binding capacity (revised Fig 3G). In the prefusion Spike structure, F1148 mediates key hydrophobic interactions within the SH trimer (revised Fig 3B), and hydrophilic substitutions that disrupt these interactions may partially compromise overall Spike folding, as the reviewer hypothesized. We have added a discussion of this point in the revised manuscript and that future structural studies of these mutants may help clarify this issue. Given that previously reported anti-S2 antibody CV3-25 targeting epitopes encompassing F1148 is to trap the prehairpin intermediate, and considering our HA-replacement mutagenesis and anti-HA neutralization assays showing that HA-insertion near the F1148 did not impair ACE2 binding but inhibits membrane fusion, our findings support the conclusion that the primary role of F1148 is to mediate membrane fusion through the hydrophobic 'hydrolock' interaction demonstrated in this study.

*Revised S5 Fig. Cell surface staining of Spike-NTD^HA^ in F1148 Mutants. (A) Schematic representation experimental design of the cell surface staining of Spike-NTDHA. Spike-NTD^HA^ was expressed on the surface of HEK293T cells, followed by incubation with primary antibody (Anti-HA mAb) and secondary antibody at 4°C for 1 hours. The percentage of FITC-positive cells and the mean fluorescence intensity (MFI) was determined by flow cytometry. (B) The population of NTD^HA^-staining-positive cells was determined by normalizing the percentage of FITC-positive cells and the mean fluorescence intensity (MFI) of the Spike.F1148 mutants to that of WT (mean values ± SDs, n=3).*

2. The fusion signals of spike/ACE2 groups seem to be significantly different in Fig. S5A and S5C. In Fig. S5C, the TMPRSS2 group should be assembled into Fig. S5A and also S5B as one data set and compare them in parallel.

We appreciate the reviewer’s constructive suggestion. In accordance with this recommendation, we repeated the relevant experiments and integrated the new results with the original dataset, which are now presented in the revised S6 Fig.

*Revised S6 Fig. Establishment and characterization of cell-cell membrane fusion. (A) Quantitative analysis of Spike-mediated membrane fusion efficiency. The donor cell population expressing Spike was incubated with different types of recipient cell populations at 37℃, and the intracellular Gluc luciferase activities were determined at 24 hours post-incubation. (mean values ± SDs, n=3). (B) The fluorescence images of panel A. Incubate the donor cell population expressing Spike with the recipient cell population expressing ACE2 at 37℃, fluorescence images were acquired at 24 hours post-incubation.*

3. In line 304, p16 and Fig. 4B-C, the authors generated hydrophilic substitutions (L977G and F782G) and assessed the impact on virus entry. It is risky since the smallest glycine mutation is commonly used to increase flexibility or disrupt the secondary structure. The authors should assess the overall prefusion conformation using different antibodies in flow cytometry, not only ACE2 binding.

We appreciate the reviewer’s constructive suggestion. Consistent with the approach described in our response to Question 1, we performed quantitative flow cytometry analysis of cell-surface-expressed Spike-NTD^HA^ constructs. The Spike·L977G mutant displayed robust anti-HA reactivity, with a positive staining ratio of 75.0% and a mean fluorescence intensity (MFI) of 64.8%, comparable to the wild-type (WT) control. In contrast, Spike·F782G exhibited a markedly reduced positive staining ratio (33.5%) and MFI (33.8%) relative to WT (revised S8 Fig). As noted in our response to Question 1, these results suggest that the F782G mutation likely induces substantial conformational alterations in Spike folding, leading to impaired VLP incorporation (revised Fig 4C). Although L977G did not noticeably affect Spike assembly or VLP incorporation (revised Fig 4C), the moderate reduction in FACS staining suggests a possible conformational change that may underlie its reduced ACE2 binding (revised Fig 4D).

*Revised S8 Fig. Cell surface staining of Spike-NTD^HA^ in L977G and F782G Mutants. The population of NTD^HA^-staining-positive cells was determined by normalizing the percentage of FITC-positive cells and the mean fluorescence intensity (MFI) of the Spike mutants to that of WT (mean values ± SDs, n=3).*

**Reviewer #2:**

The authors detected secreted proteins in the supernatant using methanol precipitation, intending to represent viral proteins incorporated into VLPs. However, this method may non-selectively precipitate all secreted proteins. Since the spike sequence used in this work is from the original strain which encodes one of the least stable spike proteins, the observed S1 signal in the supernatant could result from S1 shedding from the cellular plasma membrane rather than VLP-incorporated spike. Thus, conclusions about spike assembly defects based solely on WB results of HA-tagged S1 are not sufficiently supported. The authors should also present WB data for S2 in parallel, as the levels of S1 and S2 do not always correlate, e.g., see Figure S1G and S7B. Notably, in Figure S7B, the signal of S1 was detected in the supernatant for 1205-S-SH^HA^ and 1203-S-SH^HA^, while S2 was absent, strongly suggesting S1 shedding.
Similarly, the hACE2-GFP-Fc binding assay, performed on cell surface, should be normalized to the amount of S2 on the surface to exclude the effect of S1 shedding. Without addressing this issue, the phenotypic interpretations of spike mutants across different assays may need to be reconsidered. For example, although F782 is proposed to engage the hydrophobic cavity in the early-fusion intermediate, the F782G mutation severely disrupted spike assembly on VLP, prior to formation of the intermediate. Likewise, the L977G mutation nearly abolished VLP infectivity while remaining functional in a cell-cell fusion assay. These discrepancies require further clarification.

‌We appreciate the reviewer for raising this important point. We acknowledge that a limitation of the methanol precipitation method is its non-selective precipitation of all secreted proteins in the cell supernatant, which may include shed S1 subunits originating from cell-surface-expressed Spike protein. However, we believe that these shed S1 fragments are unlikely to significantly affect our results, particularly in Western blot (WB) analyses. Supporting this conclusion, WB analysis of VLP pellets (precipitated secreted protein samples) derived from Spike-NTD^HA^ constructs carrying mutations such as F1148I (revised Fig 3E), I977D (revised Fig 4I), and C738A/C743A (revised Fig 4L) consistently showed nearly undetectable anti-HA bands corresponding to S1 fragments. This strongly suggests that the contribution of S1 shedding is substantially lower than the efficiency of Spike incorporation via SC2-VLP assembly. Consequently, we conclude that S1 shedding has a negligible impact on our interpretations.

Furthermore, our study frequently examines the phenotypes of mutations within the S2 subunit. In this context, the use of anti-S2 antibodies for WB detection poses a potential risk, as mutations may alter epitope recognition, thereby confounding data interpretation. This concern is exemplified by the F1148 mutant, where anti-S2 antibody recognition was almost completely abolished (see figure below). Therefore, when analyzing S2-subunit mutations, we consider the use of anti-S1 antibodies (such as anti-HA for detecting Spike-NTD^HA^ constructs) to be the most reliable approach for WB analysis.

***Western blotting of Spike·F1148 mutants.*** *The plasmids Spike·WT and Spike·F1148 mutants were transfected into HEK293T cells. After 24 hours, cell lysates were harvested and subjected to Western blotting assay with the indicated antibodies (IB). The values to the left of the blots are molecular sizes in kilodaltons. Mock: naive HEK293T cells without plasmid transfection.*

Additionally, we observed that S1 and S2 levels do not always correlate in certain mutants. We propose that this discrepancy arises from differences in HA-tag insertion or substitution positions, which influence anti-HA antibody recognition efficiency. For example, in revised S2G Fig, Western blot analysis demonstrates that 23-S-NTD^HA^ shows the strongest detection signal in both VLP pellets and cell lysates, indicating superior anti-HA epitope accessibility at this position rather than being due to S1 shedding. If S1 shedding were the cause, the S1 band in cell lysates would be weaker, reflecting a reduced S1 abundance due to shedding.

In revised S9B Fig, for the 1203-S-SH^HA^ and 1205-S-SH^HA^ mutants, S2 bands (IB: HA) are almost undetectable in VLP pellets. This reduction is associated with decreased anti-HA recognition efficiency, as evidenced by markedly diminished detection in cell lysates. These HA-tag replacement positions are located near the transmembrane domain (amino acid sequence: WPWYIWLGF...), where bulky residues (e.g., W, Y, F) likely cause steric hindrance, impairing antibody accessibility during Western blotting.

We fully acknowledge the importance of quantifying and normalizing cell surface Spike expression using anti-S2 antibodies in the Spike–ACE2 binding assay. However, technical limitations, specifically the inability of anti-S2 antibodies to recognize the Spike·F1148 mutant, hinder such standardization.

For the F782G mutant, Western blot analyses confirmed that it severely disrupts Spike incorporation into VLPs (revised Fig 4C). At this stage, the Spike structure is likely significantly perturbed, rendering subsequent viral entry phenotypic assays (e.g., ACE2 binding and fusion) less informative. However, based on structural analyses, we hypothesize that F782 may also contribute to the conformational rearrangements required for membrane fusion, possibly by engaging with a hydrophobic cavity in the early-fusion intermediate. These structural hypotheses will be explored in future investigations. Please also see our response to the Question 3 raised by reviewer 1.

Regarding the L977G mutant, characterization of the two reporter signals in the membrane fusion assay revealed that while Gluc activity showed no obvious difference (revised Fig 4E), Venus fluorescence shifted from syncytial to punctate patterns (revised Fig 4F). We therefore consider that L977G impairs membrane fusion, although its inhibitory effect is less pronounced than that of F1148G. As stated in the manuscript, 'The L977G mutation may perturb a membrane fusion step not fully captured by the co-culture assay, and its effect appears less pronounced than that of F1148G, which is sufficient to surpass the detection threshold of the cell-cell membrane fusion system.' (lines 345–348).

**Reviewer #3:**

1. Line 244, have you verified in the soluble SARS-CoV-2 S2P spike to see if the molecular weight is increased by F1148I ?

We appreciate the reviewer for raising this point. We acknowledge that our study did not examine the phenotype of soluble SARS-CoV-2 S2P Spike. Instead, we employed multiple independent experiments to characterize both the intracellular expression phenotype of the Spike·F1148I mutant and its assembly phenotype on SC2-VLPs. Regarding the observed increase in molecular weight of intracellularly expressed Spike caused by the F1148I mutation, we hypothesize that this may be associated with alterations in nearby post-translational modifications. Notably, Wang et al. (Theranostics, 2025 May 25;15(13):6369-6386) recently reported that lysine residue K1149 can undergo SUMOylation. We propose that the F1148I mutation could potentially enhance this post-translational modification, thereby accounting for the observed molecular weight increase. Nevertheless, this mechanistic hypothesis requires further experimental validation.

2. For the Spike-ACE2 binding assay (lines 261-263, lines 668-669), trypsin may decrease the amount of protein or destroy the structure the protein on cell surface which may affect the result of the assay. For related result, I think it’s better to use EDTA to digest the cells. And for the binding ability, it’s better to calculate MFI with different antibody concentrations, not percentage of positive cells with a single antibody dose.

We appreciate the reviewer for raising this point. We have also observed that trypsin digestion can adversely affect Spike–ACE2 binding assays. In particular, trypsin treatment markedly reduced the binding efficiency of F1148 mutants (e.g., F1148D and F1148G) (Figure below, Panels A and B). Accordingly, our methodology explicitly specifies: 'For Spike·F1148 mutants, cells were detached by gentle pipetting instead of trypsinization'. In contrast, this detrimental effect was not observed in other Spike variants (e.g., 1160-S-SH^HA^ and 1196-S-SH^HA^), where trypsinization did not significantly affect binding activity (Figure below, Panels C–F). Therefore, trypsin digestion remains suitable for these constructs.

***The effect of trypsin on Spike-ACE2 binding efficiency.*** *The binding efficiency of F1148 mutants (A and B), 1160-S-SH^HA^ (C and D), and 1196-S-SH^HA^ (E and F) was assessed. For each mutant, the cell positivity ratio in the FITC channel and mean fluorescence intensity (MFI) were determined (mean values ± SD, n=3).*

Additionally, our Spike–ACE2 binding assays utilized soluble ACE2-GFP-Fc protein rather than antibody-based staining. We have also incorporated the reviewer’s constructive suggestion regarding MFI quantification, and the revised data are now presented in the revised Figs 3G, 4D, 6D, and 6E.

*Revised Fig 3. Identification and characterization of a hydrophobic lock (hydrolock) interaction formed by F1148 /3H /CH. (G) Binding efficiency of Spike·F1148 mutants to ACE2. The binding efficiency was determined by normalizing the percentage of GFP-positive cells and the mean fluorescence intensity (MFI) of the Spike·F1148 mutants to that of WT (mean values ± SDs, n=3).*

*Revised Fig 4. Characterization of the 3H/CH hydrophobic lock (hydrolock) interaction. (D) Binding efficiency of Spike to ACE2. The binding efficiency was determined by normalizing the percentage of GFP-positive cells and the mean fluorescence intensity (MFI) of the Spike mutants to that of WT (mean values ± SDs, n=3).*

*Revised Fig 6. Neutralization potential of the regions adjacent to PFPH-1 and PFPH-3. (D-E) Binding efficiency of Spike·SH^HA^ mutants to ACE2 (Panel D and E are 1160-S-SHHA and 1196-S-SHHA, respectively). The binding efficiency was determined by normalizing the percentage of the mean fluorescence intensity (MFI) and GFP-positive HEK293T-Spike·SHHA cells incubated with anti-HA mAb, IgG (Negative control) and XG005 (Positive control) to that of HEK293T-Spike·SH^HA^ incubated with medium as a control (mean values ± SDs, n=3).*

3. Line 498, in figure 3H, compared with WT, the hydrophilic mutations can lead to ~50% decrease, I think the change is large, maybe for the mutations, the binding affinity is not enough to trigger the following conformational change, so the fusion is decreased in figure 3J, maybe not due to the deficiency of interaction between F1148 and 3H/CH cavity, do you think it is possible?

We appreciate the reviewer for raising this point. We acknowledge the reviewer’s valid concern that reduced Spike–ACE2 binding efficiency could potentially impact membrane fusion. To address this, we performed additional experiments using a concentration gradient of XG005 on wild-type Spike (Spike·WT), simultaneously measuring Spike–ACE2 binding and membrane fusion efficiency under conditions where binding was reduced to approximately 50%. As shown in the data below, even when binding efficiency was inhibited by ~50%, membrane fusion remained largely preserved at ~70%. These results indicate that a 50% reduction in binding does not significantly impair membrane fusion. Therefore, we conclude that the abolished fusion activity in the F1148 hydrophilic mutants arises from intrinsic defects in the fusion machinery rather than their ~50% decrease in binding efficiency.

***The effect of XG005 monoclonal antibody on binding and fusion.*** *The efficiency of Spike-ACE2 binding and membrane fusion following treatment with XG005 monoclonal antibody at different concentrations was determined, with normalization to the control treated with medium (mean ± SD, n=3). Curve fitting was performed using GraphPad software, where the red and blue curves represent binding and fusion efficiency, respectively. The red dashed line denotes the antibody concentration at which binding efficiency reaches 50%, and the blue dashed line denotes the fusion efficiency at this concentration.*

**Part III – Minor Issues: Editorial and Data Presentation Modifications**

**Reviewer #1:**

1. In line 144, p8, “residues 1135-1273” were used for Alphafold prediction, which is not consistent with “AF3 prediction” in Figure 1C. The prediction region and real model 6XR8 should have some overlap.

We appreciate the reviewer for raising this point. We have now showed the previously obscured overlapping regions, as shown in the revised Fig 1B.

2. In line 147, p9, the alignment yielded a RMSD value, please specify the residue range used for the alignment.

We appreciate the reviewer for raising this point. We have now explicitly specified the residue range used for structural alignment accordingly.

3. In line 179-180, p10, please carefully check the description “as assessed by IB:S2”, which appears two times here. Please correct it accordingly.

We apologize for this oversight and have corrected accordingly.

4. In Figure 3D, please indicate whether the real density or surface display mode is shown for the model.

We appreciate the reviewer for raising this point. In response to this specific question, we believe the reviewer is referring to revised Figs 3B and 3C. We have added the corresponding description accordingly.

6. Since F1148I mutant exhibited a higher molecular weight than that of WT (Fig. 3E), did the authors check the whole coding sequence for this construct, even the complete plasmid sequence?

We appreciate the reviewer for raising this point. We performed full-length coding sequencing of plasmid Spike·F1148I and confirmed its consistency with the theoretical sequence.

7. The properties of amino acids are well known in Fig. 3F, which should be removed or put into supplementary.

We thank the reviewer for this suggestion. Following your guidance, we have removed this result.

8. Since Mut 22 also reduced the VLP infectivity by over 10-fold, the data for Mut 22 should also be included in Figure 2D.

We thank the reviewer for this suggestion. As Mut22 is located within the HR2 domain, which interacts with HR1 to form the well-characterized six-helix bundle, and exhibits a substantially weaker reduction in infectivity compared to Mut9, our study primarily focused on Mut9.

9. A careful language edit is recommended in this manuscript.

We thank the reviewer for this suggestion. We have thoroughly revised and edited the manuscript accordingly.

**Reviewer #2:**

1. The address of the author Yahan Lei is missing.

We apologize for this oversight and have added the address accordingly.

2. Lines 139-141: “The resolved structure of prefusion and … CT domains” is an incomplete sentence and should be revised.

We apologize for this oversight and have revised the sentence accordingly.

3. Line 152: the correct notation should be “PFPH3 (1179-1193aa)”.

We thank the reviewer for this suggestion and have revised the description as suggested.

4. In Figure 1A, please indicate the residue numbers for each domain.

We thank the reviewer for this suggestion. Following your guidance, we have annotated the residue numbers for each domain in the revised Fig 1A.

5. In Figure 1B, the HR2 helix is predicted by AlphaFold rather than derived from experimental data. Please note this in the figure. Also, consider placing Figure 1B after Figure 1C to improve logic flow.

We thank the reviewer for this suggestion. Following your guidance, we have adjusted the order of panels in the revised Fig 1 (1B→1D, 1C→1B, and 1D→1C), and labeled the secondary structure of the prefusion Spike protein in revised Fig 1D (formerly Fig 1B) as predicted by AlphaFold 3 (AF3).

6. In Figure 1C, have you attempted to predict the S2 structure using AlphaFold3? Given the stability of the postfusion state, this may directly yield a postfusion spike.

We utilized AlphaFold 3 to predict the S2 structure, which yielded a model consistent with the prefusion conformation, as illustrated below.

7. In figure 2C, please apply a consistent color scheme as used in other figures.

We thank the reviewer for this suggestion. Following your guidance, we have adopted a consistent color scheme matching other figures, as shown in the revised Fig 2C.

8. Why is mutant “mut22” missing in Figure 2D?

As Mut22 is located within the HR2 domain, which interacts with HR1 to form the well-studied six-helix bundle, and exhibits a substantially weaker reduction in infectivity compared to Mut9, our study primarily focused on Mut9.

9. In Figure 4K, the results are incomplete. Please at least show signals of S1, S2, N, Actin.

We thank the reviewer for this suggestion. Following your guidance, we repeated the experiment to show blots of S, S1, N, and Actin, as shown in the revised Fig 4L (formerly Fig 4K).

10. In Figure 5B, it should be labeled “PFPH-1”.

We apologize for this oversight and have revised the Figure accordingly.

11. In Figure 7, it should be “Late stage” rather than “Later stage”.

We apologize for this oversight and have revised the figure accordingly.

12. In Supplementary Fig.1, please specify the conditions in #1 - #22, such as the ratio of the transfected components. The phrase “a uniform design strategy” is too vague. This information would benefit other researchers working on VLP optimization.

We have compiled the detailed grouping information into the revised Supplementary Table 2 and uploaded it accordingly. We sincerely hope that these data will be valuable to the research community

13. Supplementary Fig. 2B-D appear unrelated to the core findings of the paper, though their inclusion is not problematic.

In Figs S2B–S2D, we engineered an HA tag at the N-terminus (NT) of the M protein. Immunoprecipitation assays using anti-HA magnetic beads confirmed efficient co-purification of all structural proteins (S, E, and N) with M^HA^. These results provide a characterization of the assembly state of our packaged SARS-CoV-2 virus-like particles (SC2-VLPs).

14. In Supplementary. Fig. 5, Gluc signal intensity varies considerably between panels A and C. In panel A, the fold change between positive and negative controls exceeds 100-fold, while in C panel it is less than 10-fold. This inconsistency should be addressed or explained.

We appreciate the reviewer for raising this point. We identified inconsistencies between Figs S5A and S5C, which were likely due to batch-to-batch variability. To address this, we repeated panels A and C within the same experimental batch, and the updated results are now presented in the revised S6 Fig.

**Reviewer #3:**

1. The stem helix may be only conserved among the beta-coronaviruses (line 42), not all the coronaviruses. And I have checked some spike sequences of different coronaviruses, the major hydrophobic residues F1148 you found in this paper also seems only conserved in beta-coronaviruses. So, for the sake of accuracy, I think you need to check the whole manuscript and change the description, your findings in this job may be only suited to beta-coronaviruses, not all the coronaviruses.

We appreciate the reviewer for raising this point. Upon further examination, we found that the stem helix is highly conserved only within betacoronaviruses, with F1148 being completely conserved. Therefore, following your suggestion, we have revised the scope of the conservation analysis in this study from 'Coronaviridae' to 'Betacoronavirus' and narrowed the scope of certain sequence homology analyses accordingly, such as in the revised Figs 1D, 4M, S4B, and S4C.

2. Although the writing is generally clear, there are some places which need to be corrected by a native English speaker, for example lines 47-49, 105-109 and so on. Some sentences are difficult to understand.

We apologize for the language. We have thoroughly revised and edited the manuscript accordingly.

3. Why do you define the residues 1141-1211 as stem helix? As reported in most of the published papers, the stem helix only contains residues 1139-1163, and residues 1139-1162 is available in prefusion conformation of SARS-CoV-2 spike (PDB ID: 6XR8).

We appreciate the reviewer for raising this point and apologize for the unclear description in the text. As noted by the reviewer, most published studies define the SH region as encompassing residues 1139–1163, and the segment spanning residues 1139–1162 has been structurally characterized (PDB ID: 6XR8). However, the structure of the region spanning residues 1163–1273 in the prefusion conformation remains unresolved. To address this gap, we predicted the structure of the Spike protein region covering residues 1135–1273. Structural alignment of this predicted model with the partially resolved prefusion Spike structure (residues 1-1162) showed a rod-like triple-helix conformation. In this alignment model, the triple helix begins at residue P1140 and extends to K1211, excluding the TM domain. Therefore, we define the SH region as encompassing residues 1140–1211 (revised Fig 1B). We have revised the text accordingly to clarify this point.

4. Line 151-152, typo, should be PFPH-1, PFPH1-2 and PFPH-3, “-3” is lost.

We apologize for this oversight and have corrected it accordingly.

5. For sequence alignment (fig 1B), “.” is always used to indicate the similar amino acids, “-” for the deficiency. And for all the panels of sequence alignment in the paper, through the numbers above the sequences, I cannot find the residues as you described in the main text. For example, F1148 cannot be found easily in figure 1B.

We appreciate the reviewer for raising this point. In the figure legend, we have detailed the methods of sequence alignment and the ways of displaying homology to enhance readability. Additionally, integer amino acid positions are labeled above the sequence alignment diagram, which allows for the quick location of amino acid positions of interest (e.g., F1148 is two positions before E1150). As shown in the revised Fig 1B (formerly Fig 1B)

Since logically we have not yet identified F1148 as a key site in revised Fig 1, we consider that it is logically inappropriate to emphasize F1148 alone here, instead, we have added the amino acid range where PFPH is located below the diagram to improve comprehension.

6. Line 571-572, 0.096 μg + 0.1952 μg + 0.926 μg + 1.12 μg do not equal to 4 μg, please check. And some of the methods are described too simply. For example, for the method of western blotting (line 599) and description in lines 178-181, through these sentences, I know you can use the anti-HA antibody to detect S1 with HA insertion, but I felt a little confused that how you could detect the S2. After I checked the “Antibodies and peptide” (line 581), I think you may use this anti-S2 antibody (GeneTex, GTX632604) to conduct the WB. The similar question for figure S7B, before I found the RBD antibody (GeneTex, GTX635692) in the “Antibodies and peptide” section, I also felt a little confused on how to detect RBD without HA insertion. As I remember, most RBD antibodies recognize spatial conformation, not linear epitopes, may be not used to do WB. I think you may need to describe directly in the method, use which antibody to detect which fragment, it may be easier to be understood for the readers. Not only the WB methods, please check all the other methods to make sure that it’s more detailed.

We apologize for this oversight. We acknowledge that there was indeed an error, and we have made corrections, revising “0.096 μg + 0.1952 μg + 0.926 μg + 1.12 μg” to “0.0192 μg S, 1.96 μg M-E, 0.92 μg N·S202R, and 1.12 μg Fluc-PS9” (line 581). Additionally, we have revised the descriptions of some Western blot experiments in the text to specify the protein fragment detected by each antibody for clarity. For the anti-RBD antibody (GeneTex, GTX635692), the manufacturer did not specify its exact recognized epitope, therefore, we are uncertain whether it recognizes a conformational epitope, but the datasheet explicitly states its validation for Western blotting.

7. Line 217, “S1147, F1148, and K1149” may be better.

We appreciate the reviewer for this suggestion. Following your guidance, we have revised “S1147F1148K1149”to “S1147, F1148, and K1149” in line 221

8. Figure 3J & H, dose F1148W and F1148M affect the binding and fusion?

We appreciate the reviewer for raising this point. To investigate the impact of the F1148W and F1148M mutations on Spike-ACE2 binding and membrane fusion, we performed additional experiments, the results demonstrate that these hydrophobic mutants ‌virtually unaffected‌ binding or fusion efficiency, as shown in the figure below.

***The effects of the F1148W and F1148M mutants on Spike-ACE2 binding and membrane fusion. (A)*** *Binding efficiency of Spike·F1148 mutants to ACE2. The binding efficiency was determined by normalizing the percentage of GFP-positive cells* ***and the mean fluorescence intensity (MFI)*** *of the Spike·F1148 mutants to that of WT (mean values ± SDs, n=3).* ***(B)*** *Membrane fusion efficiency mediated by Spike·F1148 mutants. The fusion efficiency was determined by normalizing the luciferase activity of the HEK293T-Spike·F1148 mutants to that of WT (mean values ± SDs, n=3).*

9. Line 315, it’s better to put the data in the supplemental figures.

We appreciate the reviewer for this suggestion. Following your guidance, we have presented the relevant data in the revised Fig 4E.

*Reviesd Fig 4. Characterization of the 3H/CH hydrophobic lock (hydrolock) interaction. (E) Membrane fusion efficiency mediated by Spike·L977G. The fusion efficiency was determined by normalizing the luciferase activity of the HEK293T-Spike·F1148 mutants to that of WT (mean values ± SDs, n=3).
10. The names of Y-axis in fig 3J and fig 4B are different, I think should be the same name.*

We appreciate the reviewer for raising this point. Based on the specific question description, we infer that what you mean is "The names of Y-axis in fig 4J and fig 4B are different". Accordingly, we have standardized the name of the Y-axis of Fig 4J in accordance with that of Fig 4B, as shown in the revised Fig 4K (originally Fig 4J).

11. In figure S7B, for some channels, why RBD can be detected, S2 with HA insertion can not?

We appreciate the reviewer for raising this point. In revised S9B Fig (originally Fig S7B), for the 1203-S-SH^HA^ and 1205-S-S^HA^ mutants, S2 bands (IB: HA) are nearly undetectable in VLP pellets. This is associated with reduced anti-HA antibody recognition efficiency, as evidenced by severely diminished detection in cell lysates. These HA-tag replacement positions are located near the TMD domain (amino acid sequence: WPWYIWLGF...), where bulky side-chain residues (e.g., W, Y, F) create steric hindrance during Western blotting, thereby impairing anti-HA antibody accessibility but have no effect on the anti-RBD antibody.

Sincerely,

Zhigang Yi
